# A Zinc‐ and Calcium‐Rich Lysosomal Nanoreactor Rescues Monocyte/Macrophage Dysfunction under Sepsis

**DOI:** 10.1002/advs.202205097

**Published:** 2023-01-03

**Authors:** Qin Zhao, Zijian Gong, Jiaolong Wang, Liangliang Fu, Jing Zhang, Can Wang, Richard J. Miron, Quan Yuan, Yufeng Zhang

**Affiliations:** ^1^ The State Key Laboratory Breeding Base of Basic Science of Stomatology (Hubei‐MOST) & Key Laboratory of Oral Biomedicine Ministry of Education School & Hospital of Stomatology Wuhan University Wuhan 430079 P. R. China; ^2^ Medical Research Institute School of Medicine Wuhan University Wuhan 430071 P. R. China; ^3^ Institute of Chemical Biology and Nanomedicine State Key Laboratory of Chemo/Biosensing and Chemometrics College of Chemistry and Chemical Engineering Hunan University Changsha 410082 P. R. China

**Keywords:** lysosomes, metal–organic frameworks, monocytes/macrophages, single‐cell RNA sequencing, zinc ions

## Abstract

Sepsis is a dysregulation of the immune response to pathogens and has high morbidity and mortality worldwide. However, the unclear mapping and course of dysregulated immune cells currently hinders the development of advanced therapeutic strategies to treat sepsis. Here, evidence is provided using single‐cell RNA sequencing from peripheral blood mononuclear cells in sepsis that pathogens attacking monocytes/macrophages disrupt their immune function. The results reveal an enormous decline in monocytes/macrophages in sepsis and chart the evolution of their impaired phagocytosis (Pha) capabilities. Inspired by these findings, nanoparticles, named “Alpha‐MOFs,” are developed that target dysfunctional monocytes/macrophages to actively (A) lift (L) Pha by the release of lysosome‐sensitive ions from a mineralized metal–organic framework (MOF). Alpha‐MOFs have good stability and biosafety in peripheral blood and efficiently targeted monocytes/macrophages. They also release calcium and zinc ions into monocyte/macrophage lysosomes to promote the Pha and degradation of bacteria. Taken together, these results suggest that Alpha‐MOFs rescue monocytes/macrophages dysfunction and effectively improve their survival rate during sepsis.

## Graphical abstract

The “Alpha‐MOFs” are developed, targeting dysfunctional monocytes/macrophages to actively (A) lift (L) phagocytosis (Pha) by the release of lysosome‐sensitive ions (Ca^2+^ and Zn^2+^) from a mineralized metal–organic framework (MOF). They have good stability and biosafety in peripheral blood, and also release ions into monocyte/macrophage lysosomes to promote the Pha and degradation of bacteria, which effectively improved the survival on sepsis.

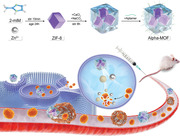

## 1 Introduction

Sepsis manifests as a dysregulation of anti‐infective processes and organ dysfunction triggered by pathogenic invasion, and the mortality rate 3 days after onset is as high as 70%.^[^ ^1^, ^2^ ^]^ Current treatment strategies mainly include supportive therapy (vasopressors, insulin infusion, etc.) and antibiotics. However, the mortality rate of septic shock is still high. Early initiation of treatment is critical in the development of sepsis, as delays may lead to multiple organ dysfunction.^[^ ^3^, ^4^ ^]^ Compelling evidence indicates that the excessive pathogen burden involved in sepsis induces ineffective immune supervision and hematogenous multisystemic infection, which is the main reason for the increased multiorgan susceptibility and high mortality.^[^ ^5^, ^6^, ^7^ ^]^ Specifically, when pathogens enter the body, monocytes/macrophages can swallow and destroy the invading pathogens, and present microbial antigens on their surfaces, leading to further adaptive immune response.^[^ ^8^, ^9^ ^]^ Therefore, the successful endocytosis and clearance of bacteria by monocyte/macrophage is the key to innate immune response. If the response to pathogenic bacteria infection in this step is not sufficient, the immune system will be more difficult to prevail in the subsequent cascade reaction of sepsis, and eventually lead to immunosuppression.^[^ ^10^, ^11^ ^]^ However, there is no clear mapping and trajectory of dysregulated immune cells during sepsis, which hinders the development of advanced therapeutic strategies.

Here, using a single‐cell RNA sequencing (scRNA‐seq) approach, we comprehensively resolved each cell population of mouse peripheral blood mononuclear cells (PBMCs) in sepsis model and healthy controls. The aim was to uncover evidence that pathogens attacking target cells disrupt immune regulation and supervision. Our findings first revealed the enormous decline in monocyte functions under sepsis and charted the evolution of impaired phagocytosis (Pha) by monocytes. Additionally, monocytes/macrophages attacked by pathogens exhibit broader and more intense intercellular communication, which may provide evidence for multiple immune cell dysregulation during sepsis.^[^ ^4^ ^]^ While many research efforts have attempted to develop immunotherapies for sepsis,^[^ ^12^, ^13^ ^]^ precisely targeting monocytes/macrophages and re‐engaging their positive functions after destruction by bacteria is still a considerable challenge. Our data suggest a potential inhibition of bacterial uptake and lysosomal degradation by monocytes/macrophages during sepsis, leading to excessive lysosomal load, whereby bacteria hiding in lysosomes utilize dysfunctional monocytes/macrophages as hosts to evade normal immune cell function and cytokines.^[^ ^14^, ^15^ ^]^ Dysfunctional monocytes/macrophages may potentially even assist bacteria in evading immune clearance, which may be a key obstacle to the treatment of sepsis. Therefore, a reprogramming strategy targeting monocyte/macrophage lysosomes was proposed to improve the function of dysfunctional monocytes/macrophages and provide a favorable opportunity for immunotherapy of sepsis.

**Scheme 1:**
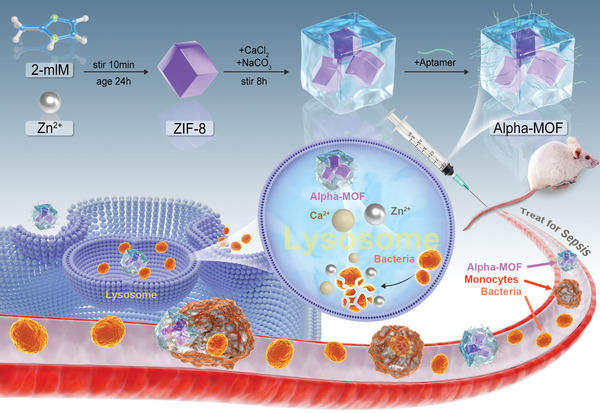
Schematic diagram of the design and synthesis of Alpha‐MOF and its function.

Inspired by this, we developed a nanoparticle system that actively targets normal and dysfunctional monocytes in the peripheral blood of sepsis model and regulates their ability to perform the Pha and lysosomal degradation of bacteria. The key to realizing this concept further was to address the stability, biosafety, and monocyte targeting ability of nanoparticles in peripheral blood circulation, as well as lysosomal responsiveness within monocytes.^[^ ^16^ ^]^ The application of metal–organic frameworks (MOFs) in medical fields such as drug delivery and antibacterial has received increasing attention in recent years, and several controlled drug release systems have been developed.^[^ ^17^, ^18^, ^19^ ^]^ However, MOF materials alone have no targeting ability and can only be compensated by large‐dose administration. At the same time, a large part of nanoparticles will be trapped by the mononuclear‐phagocytic cell system in vivo and become ineffective. In this study (**Scheme** **1**), we designed nanoparticles that satisfy the above description: lysosomal pH‐sensitive ZIF‐8 MOFs subjected to CaCO_3_ mineralization and monocyte/macrophage aptamer modification on their surface. These nanoparticles have enhanced stability and biosafety because of the mineralization on their framework. They also inherit the acid‐sensitive ion release of ZIF‐8 MOFs and CaCO_3_, enabling the release of calcium and zinc ions in lysosomes after monocyte/macrophage targeting.^[^ ^17^, ^20^, ^21^, ^22^, ^23^ ^]^ Through the action of these ions, the nanoparticles actively (A) lift (L) the Pha of monocytes/macrophages; thus, the nanoparticles were named “Alpha‐MOFs.” During treatment, Alpha‐MOFs rescued monocyte/macrophage dysfunction and effectively removed bacteria to improve survival rates during sepsis.

## 2 Results and Discussion

### 2.1 Changes in Monocyte Function in Physiological and Sepsis Settings

**Figure 1:**
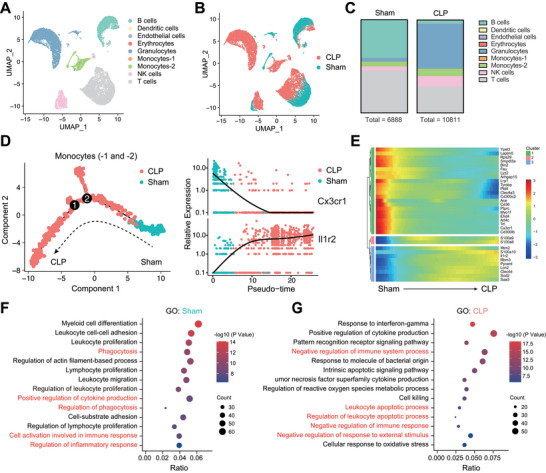
Changes in monocyte function due to sepsis. A,B) Uniform manifold approximation and projection (UMAP) plot of total blood cells isolated from normal or septic mice. Data was obtained from Gene Expression Omnibus (GSM5857054 and GSM5857055). C) Bar graph showing the proportion of cells in each group. D) Pseudotime and single cell trajectory analysis by Monocle. E) Heatmap of representative pseudotime‐dependent genes. F,G) Bubble plot showing enriched GO‐BP terms for two groups of monocytes.

To determine what changes occur in immune cells, particularly monocytes, during sepsis progression, we first analyzed a publicly available set of scRNA‐seq data. In this dataset, PBMCs from normal mice (Sham) and septic mice (CLP) were used for scRNA‐seq. We performed a basic bioinformatics analysis (Figure S1, Supporting Information), annotating individual clusters based on the MouseRNAseqData dataset into a total of nine clusters of cells (**Figure** **1**A–C). Subsequently, we collectively refer to both the cluster monocytes‐1 and ‐2 (including monocytes and macrophages) in the subpopulation as monocytes. Next, we focused on the differences between monocytes in the Sham and CLP groups (Figure S2, Supporting Information). We further constructed unsupervised single‐cell trajectories using Monocle and found a distinct boundary between the two groups of monocytes (Figure 1D). Moreover, the heatmap demonstrated the results of the pseudotime analysis of representative differential genes, reflecting the reversal of some functions of the two groups of cells (Figure 1E). Gene Ontology (GO) analysis of the differentially expressed genes (DEGs) between the two groups showed that the Sham group had a higher level of Pha and immune response, among other functions (Figure 1F). In contrast, in the CLP group, immunomodulatory functions were substantially suppressed, and even higher levels of apoptosis were observed (Figure 1G). This suggests that during sepsis, monocytes change from cells with strong positive immunomodulatory potential, such as Pha of bacteria, to dysfunctional cells that do not produce an effective immune response. We also applied CellChat to analyze the cell interaction of major immune cells in peripheral blood.^[^ ^24^ ^]^ During sepsis progression, monocytes displayed significantly enhanced cellular signals and cell‐to‐cell interactions (Figure S3, Supporting Information), which provide data support for further research on the specific mechanisms mediating monocyte dysfunction.

### 2.2 Synthesis and Characterization of Alpha‐MOFs

**Figure 2:**
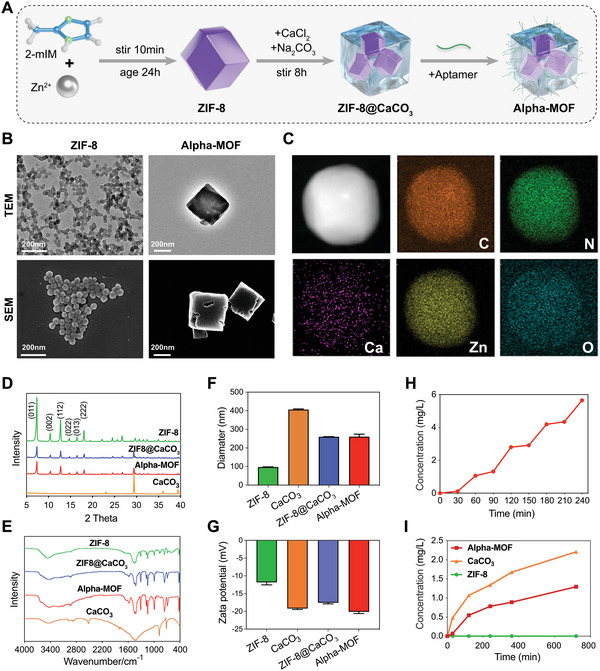
Preparation and characterization of Alpha‐MOF. A) Schematic diagram of the design and synthesis of Alpha‐MOF. B) Morphology assessment of ZIF‐8 and Alpha‐MOF using transmission electron microscopy (TEM) and scanning electron microscopy (SEM). C) EDS images showing the distribution for the elemental mapping of calcium (purple), carbon (orange), oxygen (cyan), zinc (yellow), and nitrogen (green) in Alpha‐MOF. D) The XRD patterns of ZIF‐8, ZIF‐8@CaCO_3_, Alpha‐MOF, and CaCO_3_, respectively. E) The Fourier transform infrared spectroscopy analysis. F,G) DLS measurements of hydrodynamic size (diameter) and zeta potential of NPs. Data presented as mean ± s.d. (*n* = 3). H) Zinc ion release curves of Alpha‐MOF under different pH environment (pH = 7.4/pH = 5.5) cycles. I) Calcium ion release curve of the nanomaterials at pH = 5.5.

In the synthesis of Alpha‐MOFs, we first synthesized a pH‐sensitive MOF (ZIF‐8) following the synthetic protocol using zinc acetate solution and organic ligand 2‐methylimidazole while stirring for 10 min at room temperature followed by aging for 24 h.^[^ ^25^ ^]^ Then, a calcium carbonate (CaCO_3_) coating was deposited on the surface of ZIF‐8 to maintain the acid degradability of ZIF‐8 and enhance its biocompatibility and stability. Finally, the biotin–streptavidin system was used to couple the mineralized ZIF‐8 with Pha‐Apt (aptamer which target phagocytes), enabling Alpha‐MOFs to target monocytes/macrophages. After the execution of the above process, the Alpha‐MOFs we envisioned were synthesized (**Figure** **2**A). To confirm that the synthesized Alpha‐MOFs had an ideal structure and function, we then proceeded to characterize the material. Transmission electron microscopy (TEM) demonstrated that Alpha‐MOFs had a polygonal structure similar to ZIF‐8 and a relatively uniform and irregular CaCO_3_ coating that could be seen on its surface. Combined with scanning electron microscopy (SEM) observations, the shape of ZIF‐8 after CaCO_3_ deposition changed from the original dodecahedron to an approximately cubic nanoparticle (Figure 2B). The correct assembly of Alpha‐MOFs was verified by element mapping analysis, and the necessary elements in each layer of the structure were successfully identified. The elements zinc (Zn) and nitrogen (N) were contained in ZIF‐8, as well as the elements making up CaCO_3_: calcium (Ca), carbon (C), and oxygen (O) (Figure 2C). The energy spectrum and elemental analysis results also confirmed this point (Figure S4A, Supporting Information). The structures of the nanoparticles, including ZIF‐8, CaCO_3_, ZIF‐8@CaCO_3_, and Alpha‐MOFs, were identified through X‐ray diffraction (XRD) and Fourier transform infrared (FTIR) spectroscopy (Figure 2D,E). Furthermore, their hydrodynamic diameters were measured by dynamic light scattering (DLS), and the results showed that the diameters of ZIF‐8@CaCO_3_ and Alpha‐MOFs increased slightly, changing from 97.13 nm (pure ZIF‐8) to 260.13–260.43 nm (Figure 2F). The absolute value of the zeta potential of Alpha‐MOFs also increased slightly from −11.83 to −17.57 to −20.13 mV (Figure 2G), indicating that Alpha‐MOFs had better physical stability than the others. At the same time, the results of thermogravimetric analysis also show that Alpha‐MOFs have stronger thermal stability (Figure S4B, Supporting Information). In the design, Alpha‐MOFs will be applied to peripheral blood and target monocytes/macrophages. The approach depends on the Pha‐Apt connected to the surface of Alpha‐MOFs, as well as on long‐term stability. For this reason, we used gel electrophoresis to confirm that Pha‐Apt was attached to the surface of Alpha‐MOFs and retained stable nucleic acid expression after 36 h (Figure S4C,D, Supporting Information).

To more accurately characterize the degradation ability and product composition of Alpha‐MOFs, we compared the degradation and ion release of Alpha‐MOFs in different pH environments (pH = 5.5/pH = 7.4). The results demonstrated that the degradation rate of Alpha‐MOFs in a neutral environment is relatively slow, but once exposed to an acidic environment, they quickly degrade and release the metal ions therein (Figure 2H). Alpha‐MOFs are therefore an effective controlled release system because their degradation is based on pH as the microenvironment becomes more acidic (working at pH <7.0). Furthermore, the degradation and ion release of different nanoparticles of ZIF‐8, CaCO_3_, and Alpha‐MOFs under the same pH environment (pH = 5.5/pH = 7.4) were compared. The results showed that Alpha‐MOFs had an ion release curve similar to that of ZIF‐8/CaCO_3_ in an acidic environment (Figure 2I and Figure S4E–G, Supporting Information). The degradation process of Alpha‐MOFs in an acidic environment was further demonstrated by SEM imaging depicting their morphological changes over time (Figure S4H, Supporting Information). These results indicate that the degradation ability of Alpha‐MOFs is pH‐responsive.

The difficulties in the use of MOF materials in the field of biomedicine are mainly due to their cytotoxicity, so we used the CCK8 method to investigate the cytotoxicity of Alpha‐MOFs.^[^ ^26^ ^]^ The results showed that Alpha‐MOFs could still maintain cell viability at ≈80% at 24 h, which was much higher than that of the ZIF‐8 material (Figure S4I, Supporting Information). This may be because the CaCO_3_ coating provides better biosafety, eliminating the shortcomings of MOFs.^[^ ^18^ ^]^ This evidence suggests that Alpha‐MOFs have a well‐established acid sensitivity ion release system, combined with good biosafety and stability. We therefore successfully integrated the advantages of ZIF‐8, CaCO_3_, and aptamers. These characteristics provide a guarantee and basis for further exploration regarding the biological functions of Alpha‐MOFs and their use during the treatment of sepsis.

### 2.3 The Effect of Alpha‐MOFs on the Ability of Monocytes/Macrophages to Ingest and Degrade Bacteria

**Figure 3:**
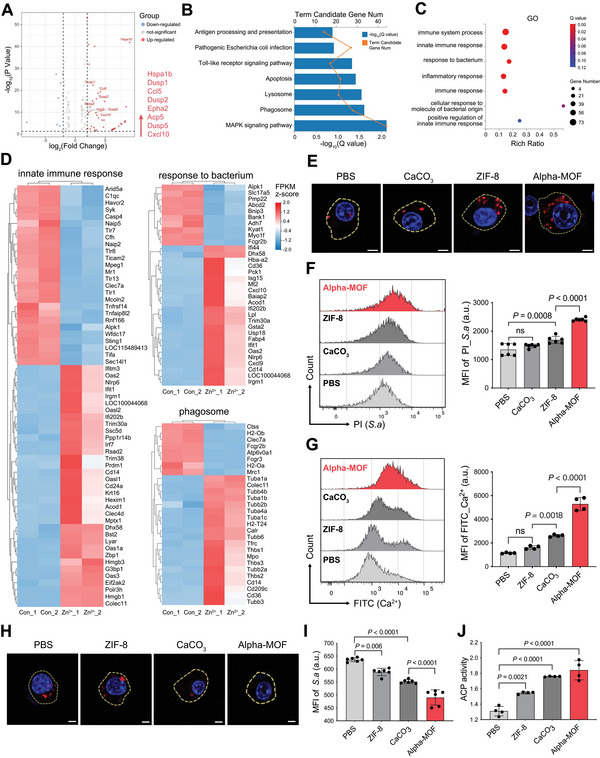
Alpha‐MOF enhances the ability of monocytes/macrophages to phagocytose and degrade bacteria. A) Volcano plot of differentially expressed genes activated by zinc ions. B) KEGG pathway analysis of upregulated genes in RAW264.7 from zinc group compared to con group. C) Gene Ontology‐biological process analysis of expressed genes (DEGs). D) Heatmap of genes, which were associated with innate immune response, response to bacterium and phagosome, ranked by differential fold change. E) Confocal fluorescence microscopy images to monitor the cellular uptake of bacteria by RAW264.7 cells. The nuclei were stained with DAPI and the bacteria were red fluorescent. Scale bars: 1 µm. F) Flow cytometry detection of bacterial phagocytosis of cells stimulated with different materials (*n* = 6). G) Detection of intracellular calcium content by flow cytometry (*n* = 4). H) Confocal fluorescence microscopy images to monitor the bacterial lysis by RAW264.7 cells. Scale bars: 1 µm. I) Flow cytometry detection of bacterial lysis ability of cells stimulated with different materials (*n* = 6). J) Acid phosphatase content of cells after application of different materials (*n* = 4).

ZIF‐8 and CaCO_3_ are essential components of Alpha‐MOFs, and they degrade to produce Zn^2+^ and Ca^2+^ in the acidic environment of lysosomes. Studies have shown that the reduction of zinc homeostasis in macrophages can impair phagocytic function and abnormal inflammatory responses. In contrast, zinc supplementation can increase the Pha of *Escherichia coli* and *Staphylococcus aureus* (*S. aureus*) by peritoneal macrophages.^[^ ^27^ ^]^ Therefore, we added zinc ions when culturing RAW264.7 cells and further explored the effect of zinc ions on the immunological process of monocytes/macrophages by RNA transcriptome sequencing technology (**Figure** **3**A). We found that the levels of phagosomes, lysosomes, autophagy, and other related pathways were significantly increased in the zinc ion‐stimulated group compared with the control group, suggesting that zinc may play an important role in promoting these aspects (Figure 3B). GO abundance analysis showed that the innate immunity, response to bacteria, and inflammatory responses of monocytes/macrophages were significantly increased after zinc ion stimulation, suggesting that zinc is also beneficial to the immunological process (Figure 3C,D). The accuracy of RNA‐seq was verified by performing RT‐qPCRs on key genes (Figure S5A, Supporting Information).

In our study, PBS, ZIF‐8, CaCO_3_, and Alpha‐MOFs were used to treat monocytes/macrophages, and their ability to take up bacteria was compared.^[^ ^28^ ^]^ The confocal fluorescence and flow cytometry results showed that cells in the ZIF‐8 and Alpha‐MOF groups phagocytosed more bacteria after 4 h of incubation with bacteria (Figure 3E,F). The sequence of Alpha‐MOF administration and the bacterial stimulation of monocytes/macrophages also affect the Pha of bacteria (Figure S5B,C, Supporting Information). This suggests that the standard degradation product Zn^2+^ of ZIF‐8 and Alpha‐MOFs may be a critical factor in enhancing the ability of monocytes/macrophages to take up bacteria. To confirm this, we first quantified the changes in intracellular Zn^2+^ after nanomaterial treatment by flow cytometry. Zn^2+^ was abundantly enriched in monocytes/macrophages after ZIF‐8 or Alpha‐MOF stimulation (Figure S5D,E, Supporting Information). Furthermore, we tested the signaling pathways related to Pha. There is evidence that the TLR4 and PI3K/AKT pathways can positively mediate Pha,^[^ ^29^, ^30^ ^]^ and their protein expression is significantly increased after treatment with ZIF‐8 or Alpha‐MOFs (Figure S5F, Supporting Information). As the proportion of Zn increased, the ability of monocytes/macrophages to take up bacteria continued to increase (Figure S5G, Supporting Information). This confirms our hypothesis that the release of zinc from ZIF‐8/Alpha‐MOFs into cells promotes the phagocytic ability of monocytes/macrophages. However, it is worth noting that excessive zinc affects MyD88‐dependent and TRIF‐dependent pathways, causing an increase in the release of inflammatory cytokines, such as TNF‐*α*, IL‐1*β*, and IL‐6. Thus, too much may hurt the body, so the dosage of nanoparticles should be strictly monitored and controlled.^[^ ^31^ ^]^

In addition, calcium ions in lysosomes play a key role in participating in signal transduction, maintaining the homeostasis of organelles, promoting lysosomal fusion, and achieving organelle acidification and are closely related to the degradation ability of lysosomes.^[^ ^32^, ^33^, ^34^ ^]^ We were surprised to observe that when the calcium ion content was higher, the treated monocytes/macrophages were more likely to degrade the bacterial cells. Specifically, we first used flow cytometry to characterize the intracellular calcium ion content. Calcium carbonate and Alpha‐MOFs significantly increased the intracellular calcium content (Figure 3G and Figure S5H, Supporting Information). However, the pure calcium carbonate group did not increase cellular calcium as efficiently as expected, possibly due to degradation in the medium. To determine the effect of Alpha‐MOFs on the ability of monocytes/macrophages to degrade bacteria, we ensured that the cells engulfed the same amount of bacteria and were then stimulated by the addition of nanomaterials. Using flow cytometry and cytofluorescence technology, it was determined that cells with Alpha‐MOFs degraded bacteria fastest (Figure 3H,I and Figure S6, Supporting Information). The calcium ion content in the lysosome was ≈5000 times higher than that in the cytoplasm. It is currently believed that calcium ions in lysosomes are necessary to fuse intracellular vesicles and lysosomes.^[^ ^35^ ^]^ The bacteria are also located in the vesicles formed by the plasma membrane after being engulfed by cells and require the participation of calcium ions for transport to the lysosome to be eliminated. When insufficient calcium ions are available, bacteria may be “trapped” in the vesicles and may even continue to proliferate. Therefore, calcium ions are essential to degrade bacteria. The degradation of bacteria by monocytes/macrophages mainly depends on the activity of various acids and phosphates in lysosomes.^[^ ^36^ ^]^ To further confirm that Alpha‐MOFs induced the biological mechanism of degrading bacteria through calcium ions, we tested the phosphatase activity regulated by Ca^2+^. The results showed that the cells treated with Alpha‐MOFs had the most potent acid phosphatase activity, which was consistent with previous results (Figure 3J).

Since calcium carbonate has the potential to neutralize the acidic environment of lysosomes, we examined monocytes/macrophages lysosomal pH after application of the material. The application of pure CaCO_3_ had a certain degree of influence on lysosomes, but Alpha‐MOFs did not have this problem (Figure S7, Supporting Information). The above results show that Alpha‐MOFs release their degradation products, Zn^2+^ and Ca^2+^, in the lysosome to promote the phagocytic ability of monocytes/macrophages and the degradation of bacteria, which complete the reprogramming of monocytes/macrophages so that they can better degrade bacteria.

### 2.4 Alpha‐MOFs Target Monocytes/Macrophages for Self‐Assembly

**Figure 4:**
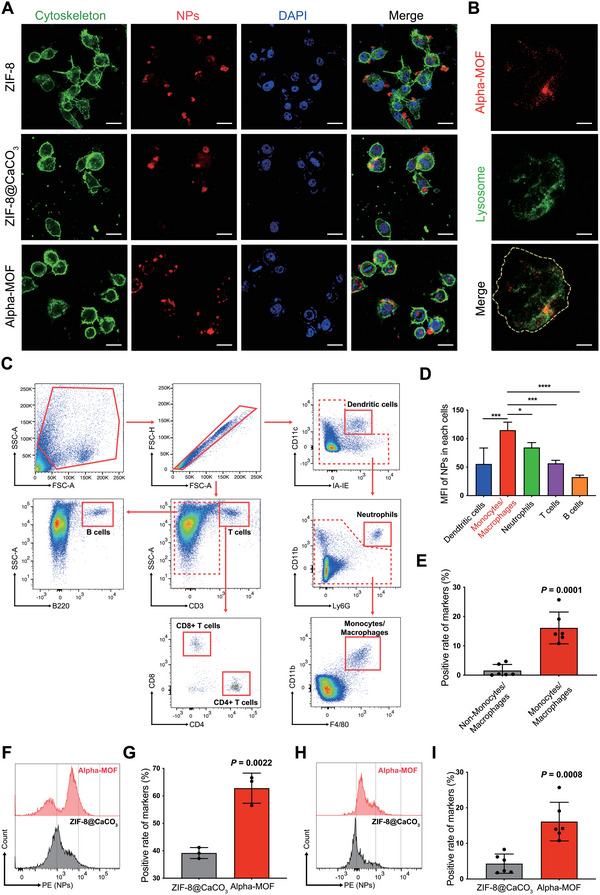
Alpha‐MOF has monocyte/macrophage targeting ability in vitro and in vivo. A) Confocal fluorescence microscopy images to monitor the uptake of NPs by RAW264.7 cells. Cytoskeleton was stained with phalloidin (green), red fluorescence was added during material preparation, and nuclei were stained with DAPI (blue). Scale bars: 20 µm. B) Confocal fluorescence microscopy images show the internalization of the Alpha‐MOF inside the cell lysosome. C) Flow cytometry gating strategy for immune cells in blood. D) Detection of MFI of several immune cells containing Alpha‐MOF materials in blood by flow cytometry (*n* = 4). E) Flow cytometry to detect the ratio of monocytes/macrophages to non‐monocytes/macrophages in the blood targeted by Alpha‐MOF (*n* = 6). F,G) Flow cytometry to detect the ability of ZIF‐8@CaCO_3_ and Alpha‐MOF to target monocytes/macrophages in in vitro blood samples (*n* = 3). H,I) Flow cytometry to detect the ability of ZIF‐8@CaCO_3_ and Alpha‐MOF to target monocytes/macrophages in vivo (*n* = 6).

Bacterial blood infection is an important clinical feature of sepsis. Targeting peripheral blood monocytes/macrophages is the first step to initiating the reprogramming of monocytes/macrophages and enhancing their abilities.^[^ ^13^, ^37^ ^]^ Pha‐Apt is the driving force of Alpha‐MOF to target monocytes/macrophages,^[^ ^38^ ^]^ and the CaCO_3_ coating is also considered to be an important factor in enhancing the affinity of nanoparticles with target cells.^[^ ^17^, ^39^ ^]^ To verify this hypothesis, we demonstrated the necessity of Pha‐Apt and CaCO_3_ coating in the synthesis through in vitro and in vivo monocytes/macrophages targeting tests. First, we cultured monocytes/macrophages with ZIF‐8, ZIF‐8@CaCO_3_, and Alpha‐MOFs and examined their targeting to monocytes/macrophages using confocal fluorescence. As shown in **Figure** **4**A, the cytoskeleton of monocytes/macrophages was labeled with green fluorescence, and the nanoparticles were labeled with TRITC (red fluorescence). After 4 h of coculture, the Alpha‐MOF group showed the highest red fluorescence in monocytes/macrophages, followed by ZIF‐8@CaCO_3_. It was discouraging to observe that pure ZIF‐8 stayed almost entirely outside of monocytes/macrophages. Alpha‐MOF targeting of monocytes/macrophages can be observed as foreign bodies entering monocyte/macrophage lysosomes, consistent with the site of bacterial degradation within the cell.^[^ ^13^, ^40^ ^]^ To determine the fate of Alpha‐MOF in cells after targeting monocytes/macrophages, we labeled the cell lysosomes with anti‐Lamp1. We found that Alpha‐MOFs (red) highly colocalized with lysosomes (green) (Figure 4B). This result is consistent with the cell TEM images (Figure S8A, Supporting Information). It needs to be emphasized that the lysosomes of monocytes/macrophages are essential organelles that take up bacteria and initiate intracellular degradation. They are also an ideal hiding place for bacteria to avoid being attacked by extracellular antimicrobial substances in the event of a severe infection.^[^ ^13^, ^14^, ^15^ ^]^ Therefore, the ability of Alpha‐MOFs to target monocyte/macrophage lysosomes is an important prerequisite for modulating lysosomal function and achieving intracellular antibacterial activity. In fact, Alpha‐MOFs enter and degrade in lysosomes, which provides a spatial basis for their further regulation of lysosomal function and antibacterial therapy.

An aptamer is a short single‐stranded nucleic acid sequence that can bind to the target molecule like an antibody. It has the characteristics of high stability, high affinity, and high specificity. It is widely used in drug delivery, biosensing, and disease diagnosis.^[^ ^41^, ^42^, ^43^ ^]^ Meilyn et al. used cell‐SELEX technology to discover an aptamer that specifically targets monocytes/macrophages and has potential to be combined with nanoparticles to target monocytes/macrophages.^[^ ^38^ ^]^ The CaCO_3_ coating also enhances the affinity of nanoparticles. This may be because the formation of CaCO_3_ makes the surface of the nanoparticles negatively charged, facilitating passage through the cell membrane.^[^ ^44^ ^]^ Here, the design of Alpha‐MOFs integrates the respective advantages of Pha‐Apt and CaCO_3_ coating, which provides sufficient and necessary conditions for a good monocyte/macrophage targeting design. Thus, to verify the targeting and specificity of Alpha‐MOFs to monocytes/macrophages in mice, we carried out tail vein injections of nanoparticles. Then, we extracted peripheral blood from mice and confirmed by flow cytometry that Alpha‐MOFs preferentially target CD11b+ peripheral blood phagocytes, including monocytes/macrophages (CD11b+F4/80+) and neutrophils (CD11b+Ly6G+), over including dendritic cells (CD11c+IA/IE+), T cells (CD3+), and B cells (B220+) (Figure 4C–E and Figure S8B, Supporting Information). At the same time, flow cytometry results showed that Alpha‐MOFs were more easily taken up by peripheral blood monocytes/macrophages than ZIF‐8@CaCO_3_, which have no aptamer attached (Figure 4F–I). The above results indicate that Alpha‐MOFs are well designed through the combination of Pha‐Apt and CaCO_3_ coating on ZIF‐8 and exhibit excellent monocyte/macrophage targeting and specificity in vitro and in vivo.

Alpha‐MOFs targeted at monocytes/macrophages can be regarded as foreign bodies entering monocytes/macrophages, which are preferentially transported to lysosomes for subsequent treatment. It should be emphasized that lysosomes of monocytes/macrophages are the basic organelles that ingest bacteria and initiate intracellular degradation. They are also ideal for bacteria to avoid the attack of extracellular antibacterial substances. Therefore, the ability of Alpha‐MOFs to target lysosomes of monocytes/macrophages is an important prerequisite for regulating lysosomal function and achieving intracellular antibacterial activity. To date, Alpha‐MOFs can actively target and reprogram monocytes/macrophages, and the overall function of Alpha‐MOFs has been shown to be complementary and necessary.

### 2.5 Alpha‐MOFs Targeted the Reprogramming of Monocytes/Macrophages in the Treatment of Blood‐Borne Sepsis and Local Necrotizing Fasciitis

**Figure 5:**
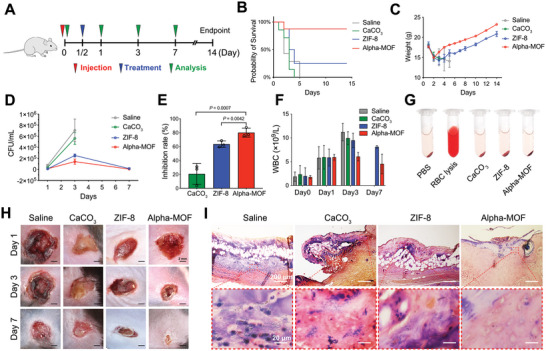
Therapeutic effect of Alpha‐MOF in sepsis and local infection models. A) Mouse sepsis model establishment and treatment schedule. B) Survival curves of mice receiving different treatments (*n* = 7–8). C) Body weight curve of septic mice (*n* = 7–8). D) Blood bacterial load curve of septic mice (*n* = 3). E) Inhibitory rate of each material on bacteria in septic mice (*n* = 3). F) The number of leukocytes in septic mice (*n* = 3). G) Photos of the material hemolysis test. H) Images of wound size change after material treatment in mouse local infection model. Photos are 1 cm long and wide. I) Modified Gram‐stained images of localized infection sites in mice at day 3. Bacteria appear as purple dots.

Considering that Alpha‐MOFs can target and strengthen the bactericidal ability of monocytes/macrophages in vitro, we next carried out in vivo treatment experiments on mice with *S. aureus*‐induced sepsis. A total of 1 × 10^7^ colony‐forming units (CFUs) *S. aureus* was injected into the abdominal cavity of mice to construct a blood‐borne sepsis model, and saline, CaCO_3_, ZIF‐8, and Alpha‐MOFs were injected through the tail vein 12 h later (**Figure** **5**A). The survival curve results showed that the untreated mice all died within 5 days, while the Alpha‐MOF group had the highest survival rate (Figure 5B). However, the development of sepsis can cause changes in the body weight of mice, which can also reflect the effect of treatment.^[^ ^13^ ^]^ We regularly tested the body weight of mice in each group, and the results showed that without effective treatment, the body weight of septic mice gradually decreased until death. However, the weight of mice in the Alpha‐MOF treatment group had the fastest recovery rate, indicating that the adverse effects of bacteria in the body were gradually restrained (Figure 5C). Since death caused by sepsis is related to the excessive proliferation of bacteria in the body, we also measured the bacterial CFU (Figure 5D,E and Figure S9A, Supporting Information) and routine blood counts (Figure 5F and Figure S9B–D, Supporting Information). Compared with saline and CaCO_3_ treatment, both ZIF‐8 and Alpha‐MOF treatment reduced the bacterial load in the blood. However, in terms of survival rate, Alpha‐MOFs had the best performance.

Biosafety is an extremely important point for the application of MOF‐related materials in medicine, and many tests have also been conducted. The healthy mice received tail vein injections of saline, CaCO_3_, ZIF‐8, or Alpha‐MOFs. We measured routine blood counts and biochemical indicators and performed HE staining on the main organs 7 days later (Figure S10A–D, Supporting Information). The results showed that different materials did not affect the levels of white blood cells (WBCs), red blood cells, or platelets in mice, and there was no apparent damage to the main organs (heart, liver, spleen, lung, and kidney). However, we noticed that ZIF‐8 treatment alone increased the AST index, while Alpha‐MOF treatment maintained ALT and AST levels in the safe range, which proved that our modification of ZIF‐8 improved its safety. Since the nanomaterials were transported directly through the blood, we tested their hemolytic ability. The results showed that there was no apparent hemolysis in any group of materials (Figure 5G).

We also simulated sepsis caused by localized necrotizing fasciitis by subcutaneously injecting bacteria into the backs of mice. Each mouse was injected with 100 µL containing 5 × 10^6^ CFU of *S. aureus* for 3 consecutive days, and then the site was injected with saline, CaCO_3_, ZIF‐8, or Alpha‐MOFs for treatment after skin ulceration. To evaluate the bactericidal effect and systemic inflammation, the blood of mice was taken on the 3rd, 7th, and 10th day after treatment for routine blood tests. The WBC level under Alpha‐MOF treatment peaked on the 7th day and then began to decline, indicating that the control of bacterial infection was initially completed. In comparison, the WBC levels in the other groups were still high (Figure S10E, Supporting Information). Figure 5H shows that as time goes by, the wound area gradually decreases, and the wound healing rate after Alpha‐MOF treatment is significantly higher than that of other groups. We also performed HE, Masson, and modified Gram staining on the wound tissues on the 3rd and 7th days (Figure 5I and Figures S11 and S12, Supporting Information).^[^ ^45^ ^]^ The Masson staining showed that there were many inflammatory tissues and cells (red) in each group at the early stage of infection (Day 3). In the later stage (Day 7), the body can complete the regeneration of collagen fibers in the skin healing process (blue).^[^ ^46^ ^]^ In this process, Alpha‐MOFs caused the smallest range of inflammation in each period, and the final skin repair effect was also the best. The modified Gram stain clearly showed *S. aureus* (purple dots) in the tissue, and Alpha‐MOFs had the best antibacterial effect at all stages.

It can be seen from the above in vivo experimental results that treatment with Alpha‐MOFs has a good clinical effect against localized necrotizing fasciitis and systemic sepsis infection caused by *S. aureus*. Alpha‐MOFs can effectively control the body's inflammatory response, exert antibacterial effects to promote wound healing, and rescue the adverse consequences of sepsis infection. At the same time, we also proved that it has good biosafety. In summary, Alpha‐MOF provides a promising treatment for bacterial infections such as sepsis.

## 3 Conclusion

We provide a comprehensive atlas of PBMCs during sepsis by using scRNA‐seq analysis to reveal monocytes/macrophages with impaired Pha and bacterial degradation. Importantly, we provide strong evidence for aberrant trajectories and communication of dysfunctional monocytes/macrophages in peripheral blood. Therefore, Alpha‐MOFs were designed, using a mineralized MOF to overcome the problems of poor stability and high clearance of nanoparticles in peripheral circulation; using an aptamer to target monocytes/macrophages in peripheral blood; and using the pH sensitivity of nanoparticles to release calcium and zinc ions in lysosomes. Through the action of these ions, Alpha‐MOFs actively boost the Pha and degradation of monocytes/macrophages, rescuing monocytes/macrophages dysfunction and improving the survival rate in sepsis. Overall, Alpha‐MOFs may provide an applicable strategy for curing patients with sepsis or other serious bacterial infections.

## 4 Experimental Section

### Chemicals and Reagents

2‐Methylimidazole was purchased from Sigma‐Aldrich. Avidin was bought from Shanghai Yuanye Biological. Zinc acetate, sodium carbonate, calcium chloride, cyclohexane, Triton X‐100, 1‐hexanol, albumin, glutaraldehyde, and all other reagents were purchased from Sinopharm Chemical Reagent Co. Ltd. and of analytical grade. Tetramethyl rhodamine‐labeled BSA (BSA‐TRITC) was synthesized by GuoPing Pharmaceutical Co., Ltd. Aptamers were all synthesized by Sangon Biotech. The synthesized aptamers were purified by HPLC.

Pha‐Apt‐Biotin: 5′‐GAAGAGTAGATGAAACGTTTTTTCGCCCGATAAAAGGGACGTGCGTCAGACA‐3′biotin

### Characterization

The morphologies of nanomaterials were recorded with a TEM with a working voltage of 200 kV (JEM‐2100, JEOL). The SEM images were obtained on a field‐emission SEM (Zeiss SIGMA). The average particle size and Zeta potential were measured with Zetasizer Nano ZS90 (Malvern). XRD patterns were collected on an XPert Pro (PANalytical B.V.) at room temperature. FTIR spectroscopy was carried out on FTIR5700 (Thermo Scientific). Thermal Analysis was conducted by TGA2/DSC3 (Mettler‐Toledo). The concentrations of zinc ion and calcium ion were determined by atomic absorption spectrometry (contrAA700, Analytik Jena AG). TECNAI G2 F30 TEM was used for element mapping test.

### Data Collection and Processing of Single‐Cell Transcriptome Sequencing

Publicly available scRNAseq datasets were obtained from Gene Expression Omnibus DataSets (GSM5857054 and GSM5857055). Downstream analysis steps were performed with “Seurat.” For quality control, cells with a percentage of mitochondrial genes below 10% and between 200 and 5000 genes detected were retained. Next, the matrix was normalized using the “NormalizeData()” function. Principal component analysis was performed based on the 2000 highly variable genes detected in the analysis. For the clustering of the whole dataset, the resolution was set to 0.5, respectively. In dimension reduction, pc use = 1:10. Clusters were annotated based on MouseRNAseqData dataset. The visualization of UAMP and heatmap was done by “DimPlot()” and “Doheatmap()” functions, respectively. The “FindAllMarkers()” function was used to obtain DEGs. In the R package clusterProfiler, the GO analysis of the top DEGs was performed using the “enrichGO()” function, the *p* value cutoff was set to 0.05. For pseudotime analysis, the R package monocle was used. Draw cell trajectory was done with “plot_cell_trajectory” function. When generating Pseudotime parameters, “reverse = T” was used to correct. For cell interactions, the CellChat package was used. “ComplexHeatmap,” “netVisual_circle,” “netVisual_heatmap,” and “netAnalysis_signalingRole_heatmap” functions were used to draw relevant pictures.

### Synthesis of Nanomaterials

2‐Methylimidazole aqueous solution (5 mL, 3.50 m) with zinc acetate dihydrate solution (0.5 mL, 0.50 m) were stirred for 10 min, aged for 24 h, and centrifuged with ultrapure water at 12 000 rpm for three times to obtain ZIF‐8 nanoparticles. In order to encapsulate TRITC, 2 µL of TRITC‐BSA (10 mg mL^−1^) could be added into the above solution system before stirring, and the whole process was operated under dark conditions. To prepare ZIF‐8@CaCO_3_ NPs (ZC‐NPs), cyclohexane (7.5 mL), Triton X‐100 (1.77 mL), and 1‐hexanol (1.6 mL) were first mixed thoroughly, and then, a calcium chloride solution (600 µL, 30 mm) containing ZIF‐8 NPs (20 mg) was added to form a uniformly dispersed water‐in‐oil emulsion. Finally, a sodium carbonate solution (60 µL, 2.92 m) was added, and the mixture was stirred overnight. No addition of ZIF‐8 was required when synthesizing individual CaCO_3_ microspheres. ZIF‐8@CaCO_3_ NPs were separated by centrifuging at 8000 rpm for 5 min after being washed three times with absolute ethyl alcohol. The avidin solution (50 µL, 5 mg mL^−1^) was mixed with the ZIF‐8@CaCO_3_ NPs (20 mg) and incubated for 4 h. The solution was centrifuged at 8000 rpm for 5 min, and the supernatant was removed. Next, 25% glutaraldehyde (20 µL) was mixed with the avidin‐loaded ZIF‐8@CaCO_3_ NPs for 2 h. After centrifugation and rinsing, the biotin‐modified aptamer (10 µL, 650 mm) was finally added, and the mixture was incubated overnight at 4 °C. Alpha‐MOF was separated by centrifuging at 8000 rpm for 5 min after being washed three times with ultrapure water.

### pH Sensitivity Evaluation

In order to study the release kinetics of calcium and zinc ions from nanoparticles, the nanoparticles were placed in PBS buffer solution at pH 7.4 and pH 5.5 at room temperature. The supernatant was collected by centrifugation at 10 000 rpm for 5 min at specific intervals (30 min, 2, 4, 12, and 16 h). The concentration of calcium and zinc ions in the solution was determined by atomic absorption spectrometry (contrAA700, Analytik Jena AG).

### RNA Transcriptome Sequencing

RAW264.7 cells were seeded into a 10 cm plate for 24 h. Zinc sulfate solution was added to the experimental group so that the final concentration of zinc ions was 0.1 mmol L^−1^, and the same amount of PBS was added to the control group. After culturing for 24 h, the supernatant in the dish was aspirated and gently rinsed twice with 3 mL of PBS. 2 mL Trizol was added and was let to stand on ice for 5 min, then it was collected into RNase‐free tubes, and stored in −80 °C freezer. A part was saved for subsequent PCR experiments. After the liquid was frozen, the samples were sent to BGI using dry ice for subsequent RNA extraction, library building, computerization, and analysis.

Differential gene expression analysis was conducted in DESeq2 and significant differentiation genes were identified when adjusted *p* <0.05 and log2FC ≥1. And then significant differential genes were performed for KEGG gene enrichment and GO enrichment. The visualization of the results was made using Dr. Tom system (BGI‐Shenzhen, China) and Hiplot (https://hiplot.com.cn).

### Cells and Bacteria

The RAW264.7 cell line was obtained from the American Type Culture Collection (ATCC) and cultured in Dulbecco's Modified Eagle's Medium (Gibco, Invitrogen) with 10% fetal bovine serum (Gibco, Invitrogen) at 37 °C in a humidified 5% CO_2_ atmosphere. *S. aureus* (ATCC 25923) was inoculated into LB broth at 37 °C and cultured in an aerophilic environment. All the bacteria used in the experiment were in logarithmic growth phase. Absorbance was measured by microplate reader PowerWave XS2 (BioTek, USA).

### Immunofluorescent Staining

*Nanomaterials targeting ability*: RAW264.7 cells were seeded into a 15 mm confocal culture dish at a density of 40 000 cells per dish for 24 h. Different fluorescent nanoparticles were added to the medium and incubated for 4 h. After washing with PBS for three times, cells were fixed with 1 mL 4% paraformaldehyde for 20 min, treated with 1 mL 0.1% Triton‐X‐100 for 15 min and blocked with 1 mL 1% BSA solution at 37 °C for 1 h. For lysosome‐targeting test, Anti‐LAMP antibody (200 µL, 1:200, Cat No:A16894, Abclonal) was incubated with cells at 4 °C overnight, and the secondary antibodies DyLight 405 goat antirabbit IgG (200 µL, 1:200, Cat No:A23120, Abbkine) with blue fluorescent markers were added next after washing with PBS. For monocytes/macrophages‐targeting test, 200 µL of green fluorescent phalloidin staining reagent (1:500, G1028, Servicebio) was added and incubated at 37 °C for 1 h. The cells were washed three times with PBS and mounted with DAPI (200 µL, C1002, Beyotime) for 8 min. Finally, the images were obtained by fluorescence confocal microscope (Zeiss LSM880).

*Preparation of fluorescent bacteria*: 250 µL of 2.5 × 10^6^ CFU mL^−1^ bacterial suspension was added into each well of 96‐well plate with round bottom, and centrifuged at 2500 rpm for 5 min to remove the supernatant. 4% paraformaldehyde was used for fixation for 20 min, and then centrifuged at 2500 rpm for 5 min to remove the supernatant. PI fluorescent dyes (1:20, 40755ES64, Yeasen Biotech Co., Ltd.) and DIO fluorescent dyes (1:20, C1038, Beyotime Biotechnology) were prepared to prepare red or green fluorescent bacteria, respectively. Each well was added with 100 µL dye solution, incubated at 37 °C for 15 min, and centrifuged at 2500 rpm for 5 min to remove the supernatant. Then they were washed with PBS and centrifuged twice (2500 rpm for 5 min). Finally, 200 µL PBS was used to resuspend each well and keep it at 4 °C.

*Phagocytosis/degradation of bacteria*: RAW264.7 cells were seeded into a 15 mm confocal culture dish at a density of 40 000 cells per dish for 24 h. For Pha of bacteria, different nanoparticles were added to the medium and incubated for 4 h, and then the supernatant was removed and an equal amount of fluorescent bacteria (MOI = 100:1) was added for 2 h. For degradation of bacteria, an equal amount of fluorescent bacteria (MOI = 100:1) were added to the medium and incubated for 2 h, and then the supernatant was removed and different nanoparticles were added for 12 h. After washing with PBS for three times, cells were fixed with 1 mL 4% paraformaldehyde for 20 min, treated with 1 mL 0.1% Triton‐X‐100 for 15 min, and blocked with 1 mL 1% BSA solution at 37 °C for 1 h. 200 µL of green fluorescent phalloidin staining reagent (1:500, G1028, Servicebio) was added and incubated at 37 °C for 1 h. The cells were washed three times with PBS and mounted with DAPI (200 µL, C1002, Beyotime) for 8 min. Finally, the images were obtained by fluorescence confocal microscope (Zeiss LSM880).

### Flow Cytometry

*Detect intracellular calcium/zinc ions*: RAW264.7 cells were seeded into a 6‐well plate at a density of 100 000 cells per well for 24 h. Different nanoparticles were added to the medium and incubated for 4 h. Then, the bacteria with different fluorescence were incubated for several hours. RAW264.7 cells were collected and placed in 96‐well plate with round bottom and centrifuged at 2500 rpm for 5 min to remove the supernatant. In order to determine the intracellular calcium content, the cells were incubated with fluo‐4 (50 µL each well, 1:1000, S1060, Beyotime Biotechnology) at 37 °C for 30 min. In order to determine the intracellular zinc concentration, the cells were incubated with zinc indicator (50 µL each well, 1:1000, MX4516, Shanghai Maokang Biotechnology) at 37 °C for 30 min. Then centrifugation was done at 2500 rpm for 5 min, the supernatant was removed, and resuspended in PBS, and used BD LSRFFortessaX‐20 to detect.

*Detect intracellular bacterial*: RAW264.7 cells were seeded into a 6‐well plate at a density of 100 000 cells per well for 24 h. For Pha of bacteria, different nanoparticles were added to the medium and incubated for 4 h, and then the supernatant was removed and an equal amount of fluorescent bacteria (MOI = 100:1) was added for 2 h. For degradation of bacteria, an equal amount of fluorescent bacteria (MOI = 100:1) was added to the medium and incubated for 2 h, and then the supernatant was removed and different nanoparticles were added for 12 h. Treatment was done with antibiotics to wash out extracellular bacteria. RAW264.7 cells were collected and placed in 96‐well plate with round bottom and centrifuged at 2500 rpm for 5 min to remove the supernatant. BD LSRFFortessaX‐20 was used to detect intracellular fluorescence.

### Cellular Acid Phosphatase Assay

Use cell lysis buffer for western and IP without inhibitors (P0013J, Beyotime) to initially extract protein samples to obtain cell lysate supernatant. The chromogenic substrate and standard working solution was prepared according to the ACP kit instructions (P0326, Beyotime). Each component was added in turn to a flat‐bottom 96‐well plate, and incubated in a 37 °C incubator for 30 min after mixing. 160 µL of reaction stop solution was added to each well to stop the reaction, and a microplate reader (PowerWave XS2, BioTek) was used to measure the absorbance of each well of the plate at 405 nm.

### In Vitro and In Vivo Aptamer Targeting Ability

In order to verify the function of aptamers in vitro, six C57BL/6 mice were sacrificed to obtain whole blood. Materials (0.2 mg) with or without aptamers were added to three groups of 1 mL whole blood samples and incubated at 37 °C for 4 h. As for the in vivo experiment, 200 µL of saline containing different materials (0.2 mg) was injected into each mouse through tail vein, and the mice were sacrificed 4 h later to obtain blood (1 mL for each). After obtaining whole blood containing the material in vivo or in vitro, the blood was treated with Red Blood Cell Lysis Buffer (C3702, Beyotime Biotechnology) at room temperature for 5 min, and then centrifuged at 2500 rpm for 5 min to remove red blood cells. The cells were washed twice with PBS, and divided into two parts. The first part was incubated with APC‐Cy7 anti‐CD11b antibody (1:200, Cat No: 557657, BD), PE‐Cy7 anti‐F4/80 antibody (1:800, Cat No: 4281123, BD), APC‐A700 anti‐IA/IE antibody (1:200, Cat No: 107622, Biolegend), PerCP anti‐CD11c antibody (1:200, Cat No: 117326, Biolegend), and FITC anti‐Ly6G antibody (1:200, Cat No: 127605, Biolegend) for 30 min at 4 °C. The other part was incubated with PerCP‐cy5.5 anti‐CD3e antibody (1:200, Cat No: 551163, BD), APC anti‐CD4 antibody (1:200, Cat No: 100516, Biolegend), FITC anti‐CD8a antibody (1:200, Cat No: 100706, Biolegend), and APC‐cy7 anti‐B220 antibody (1:200, Cat No: 552094, BD) for 30 min at 4 °C. The cells were then centrifuged at 2500 rpm for 5 min to remove the supernatant. The fluorescent protein in the materials could be detected under the PE channel. The flow cytometry was carried out by BD LSRFortessaX‐20.

### In Vivo Treatment of *Staphylococcus aureus*‐Induced Blood‐Borne Sepsis Mice

*S. aureus* was derived from ATCC (ATCC25923). The strains were grown on LB medium under suitable conditions (37 °C, 5% CO_2_). A single colony was picked with an inoculating loop and it was placed in a shake tube, incubated at 37 °C and 200 rpm for 8 h before use. 6‐week‐old male C57BL/6 mice were provided by Hubei Laboratory Animal Research Center. All animal experiments were approved by the Ethics Committee of School and Hospital of Stomatology, Wuhan University (ethical approval number A31‐2020). The animal experiment process complied with all relevant ethics. Sepsis model was established by intraperitoneal injection of 0.1 mL *S. aureus* bacterial suspension (1 × 10^8^ CFUs). After 12 h, 0.2 mL saline with different materials (ZIF‐8, CaCO_3_, Alpha‐MOF and control, 60 mg kg^−1^) were injected into the tail vein of mice. Body weight and survival of mice were assessed every 24 h in the 1st week and every other day in the following 7 days. Blood samples were collected from the orbital vein of the surviving mice on day 1, day 3, and day 7 to assess the amount of CFUs in the blood and blood routine. The CFUs in the blood were determined by serial dilution and plate counting.

### In Vivo Treatment of *Staphylococcus aureus*‐Induced Local Necrotizing Fasciitis Mice

The experimental animals were female C57 mice aged 6–8 weeks. The method of anesthesia was isoflurane inhalation anesthesia. The dorsal skin of the mice was removed with a shaver and further depilated with depilatory cream. The bacterial solution (5 × 10^6^ CFU) was diluted with saline to 100 µL and injected subcutaneously into the back of mice for 3 consecutive days. Skin ulceration indicated that the local infection model was successfully established, and then local injection of normal saline, ZIF‐8, CaCO3, and Alpha‐MOF was performed for treatment. Mice infection sites were photographed on days 0, 3, 7, and 10 of modeling. At the same time, orbital blood was collected for routine blood test. Mice were euthanized using carbon dioxide, and infection site tissue was clipped for subsequent staining.

### Tissue Staining

The tissue was first fixed, dehydrated, embedded, sectioned, and deparaffinized. H&E staining and Masson staining were done according to the kit instructions (G1001, G1004, Servicebio; MST‐8003, MXB Biotechnologies). For modified Gram stain (G1060, Solarbio), staining was done with crystal violet for 1 min and rinsed with water for 1 min. Staining with iodine was done for 1 min and rinsed with water for 1 min. The specimen was gently shaken for 1 min with destaining solution and rinsed with water for 1 min. Staining with safranin was done for 1 min and rinsed with water for 1 min. Dehydration was done in 95% alcohol for 3 s. The specimen was stained in alcohol saffron (1 g of saffron was soaked in 100 mL of absolute ethanol for 48 h and the filtrate was taken) stain for 30–60 s. Dehydration was done in 100% alcohol for 3 s. It was placed in a fume hood to dry. The sections were placed in xylene for transparency, and then mounted with a transparent resin. After the resin dried, the sections were photographed using a microscope.

### Hemolysis Test

200 µL of red blood cells were diluted to 1 mL with PBS, and 10 mg of each nanoparticle was added. They were incubated for 30 min in a 37 °C incubator. After centrifugation at 2500 × *g* for 5 min, the absorbance value of 540 nm of the supernatant was read using a microplate reader. A positive control was prepared by treating RBC with red blood cell lysis buffer (C3702, Beyotime Biotechnology). The hemolysis rate of each group was calculated according to the following formula:

(1) $$\mathrm{Hemolysis}\;\;\mathrm{rate}=\frac{\mathrm{Abs}\left(\mathrm{experiment}\right)}{\mathrm{Abs}\left(\mathrm{lysis}\;\mathrm{buffer}\right)}\times 100\%$$

### Biosafety Testing

6‐week‐old male C57BL/6 mice were provided by Hubei Laboratory Animal Research Center. 0.2 mL saline with different materials (ZIF‐8, CaCO_3_, Alpha‐MOF, and control, 60 mg kg^−1^) was injected into the tail vein of mice. On the 14th day, the mice were sacrificed and blood samples were collected for blood routine and biochemical tests. The internal organs (heart, liver, spleen, lung, and kidney) were removed and histologically analyzed using H&E staining.

### Additional Instructions for Single‐Cell Sequencing

A basic bioinformatics analysis was performed with quality controls, annotating individual clusters based on the MouseRNAseqData dataset into a total of nine clusters of cells. The sham group was composed of 6888 cells, and the CLP group was composed of 10 811 cells. Scatter plots and FeaturePlot clearly revealed that different cell subpopulations had unique gene expression patterns that were consistent with the biological definition of each cell subpopulation. Compared with solid tissues, the precursors of macrophages, namely, monocytes, were abundant in the peripheral blood environment, and the two were identified as homologous, demonstrating similar functions. Subsequently, both the cluster monocytes‐1 and ‐2 in the subpopulation were collectively referred as monocytes. The FindAllMarkers() function was then used to analyze the DEGs of monocytes and further enriched their GO analysis based on the DEGs. It is also known that monocyte populations played important roles in cell differentiation, migration, adhesion, and cytokine production. The monocyte subpopulations were decreased separately and analyzed the DEGs of both groups.

During sepsis progression, monocytes displayed significantly enhanced cell‐to‐cell interactions. Monocytes only sent out a large number of cellular signals in a normal microenvironment, but during sepsis, they both sent and received a large number of signals. Overall, monocytes had the highest number and intensity of cell‐to‐cell interactions during sepsis. Bubble plots showed the major ligand‒receptor pairs that mediate these cellular interactions. At the same time, changes in conserved and specific signaling pathways and overall signaling patterns were also reflected in the histogram and heatmap.

### Statistical Analysis

Unless otherwise specified, data were expressed as the mean ± SD. All experiments were repeated at least three times. For in vitro experiments, the *n* corresponded to biological replicates, and guaranteed the consistency of results in the case of technical replicates. The comparison between the two groups was performed using an unpaired two‐tailed *t*‐test. For comparison between three or more independent groups, one‐way analysis of variance was performed using Tukey's multiple comparisons test. Statistical analysis was performed using GraphPad Prism 7. When *p* <0.05, the difference was considered statistically significant.

## Conflict of Interest

The authors declare no conflict of interest.

## Supporting information

Supporting Information

## Data Availability

The data that support the findings of this study are available from the corresponding author upon reasonable request.
