# Supplementary material for: A Zinc‐ and Calcium‐Rich Lysosomal Nanoreactor Rescues Monocyte/Macrophage Dysfunction under Sepsis
Source: Adv Sci (Weinh). 2023 Jan 3;10(6):2205097. doi: 10.1002/advs.202205097 (PMC9951326; doi:10.1002/advs.202205097)
Supplement: Supplementary file 1 — Supporting Information [file ADVS-10-2205097-s001.pdf]

ADVANCED SCIENCE
Open Access

Supporting Information

for *Adv. Sci.*, DOI 10.1002/advs.202205097

A Zinc- and Calcium-Rich Lysosomal Nanoreactor Rescues Monocyte/Macrophage Dysfunction under Sepsis

*Qin Zhao, Zijian Gong, Jiaolong Wang, Liangliang Fu, Jing Zhang, Can Wang, Richard J. Miron, Quan Yuan and Yufeng Zhang**

Supporting Information

A Zinc- and Calcium-Rich Lysosomal Nanoreactor Rescues Monocyte/Macrophage Dysfunction under Sepsis

*Qin Zhao, Zijian Gong, Jiaolong Wang, Liangliang Fu, Jing Zhang, Can Wang, Richard J. Miron, Quan Yuan and Yufeng Zhang**

Dr. Q. Zhao, Z. Gong, Dr. J. Wang, L. Fu, J. Zhang, C. Wang, Prof. R. J. Miron and Prof. Y. Zhang

The State Key Laboratory Breeding Base of Basic Science of Stomatology (Hubei-MOST) & Key Laboratory of Oral Biomedicine, Ministry of Education, School & Hospital of Stomatology, Wuhan University, Wuhan 430079, China

Medical Research Institute, School of Medicine, Wuhan University, Wuhan, 430071, China

E-mail: zyf@whu.edu.cn

Prof. Q. Yuan

Institute of Chemical Biology and Nanomedicine, State Key Laboratory of Chemo/Biosensing and Chemometrics, College of Chemistry and Chemical Engineering, Hunan University; Changsha, 410082, China.

Keywords: scRNA-seq, metal-organic framework, zinc ions, monocyte/macrophage, lysosome

Methods

Nucleic Acid Electrophoresis

2μl 6x DNA loading buffer (D0071, Beyotime Biotechnology) was added into 10 μl PBS containing different materials and mixed well. The electrophoresis was carried out with 1% agarose gel, and the voltage was set to 197V for 10 minutes.

Real-time Quantitative polymerase chain reaction

200 μl of chloroform was added to 1 ml of Trizol in which the samples were stored, and the supernatant was collected after shaking and centrifugation. An equal amount of isopropanol was added, and the supernatant was discarded after centrifugation. Wash twice with 75% alcohol and dissolve in DEPC water. Concentrations were measured using nanodrop. Obtain cDNA according to reverse transcription instructions(Q712, Vazyme). The reaction system was configured according to the qRT-PCR instructions(R323, Vazyme) and tested using CFX connect(Bio-rad). The primer sequences are shown in Table 1

Table 1

Gene	Primer sequences (F, forward; R, reverse)
Hspa1b	F: 5'-ACTTGATAGCTGCTTGGGCA-3'
	R: 5'-ACAGTGCTGCTCCCAACATT-3'
Dusp1	F: 5'-TGTTGTTGGATTGTCGCTCCT-3'
	R: 5'-TTGGGCACGATATGCTCCAG-3'
Ccl5	F: 5'-GCTGCTTTGCCTACCTCTCC-3'
	R: 5'-TCGAGTGACAAACACGACTGC-3'
Epha2	F: 5'-GCACAGGGAAAGGAAGTTGTT-3'
	R: 5'-CATGTAGATAGGCATGTCGTCC-3'
Dusp2	F: 5'-TGTGGAAATCTTGCCCTACCT-3'
	R: 5'-CCCACTATTCTTCACCGAGTCTA-3'
Acp5	F: 5'-CACTCCCACCCTGAGATTTGT-3'
	R: 5'-CCCCAGAGACATGATGAAGTCA-3'
Dusp5	F: 5'-GAAGTGCCTACCACGCATCC-3'
	R: 5'-TCCGGCGGGAAACATTCAG-3'
Cxcl10	F: 5'-CCAAGTGCTGCCGTCATTTTC-3'
	R: 5'-GGCTCGCAGGGATGATTTCAA-3'
H2-T24	F: 5'-GGTCGCACTCTCTGCATTACT-3'
	R: 5'-GCCACTGGCGAAAAATGAAGG-3'

Gapdh	F: 5’-AGGTCGGTGTGAACGGATTTG-3’
	R: 5’-TGTAGACCATGTAGTTGAGGTCA-3’

Cytotoxicity test:

5×10^3 RAW264.7 cells were seeded into a 96-well plate overnight and then incubated with different nanoparticles for 12 or 24 hours. Cell viability was measured using the CCK8 kit (BS350A, Biosharp) according to the instructions. Briefly, after removing the old medium, 20μl of CCK8 reagent was added into the 200μl of medium to each well and incubated for 2 hours in a 37 °C carbon dioxide incubator. The absorbance of a 96-well plate at 450 nm was read using a microplate reader. Cytotoxicity was calculated using untreated cells as a control.

Western Blot

RAW264.7 cells were seeded into a 6 well plate at a density of 100,000 cells per well for 24 hours. Different nanoparticles were added to the medium and incubated for 36 hours. Cells were treated with RIPA lysate (P0013B, Beyotime)containing PMSF(ST507, Beyotime) and phosphatase inhibitors(Roche). Sonicate the lysate until it is relatively fluid. The supernatant was collected by centrifugation at 12000r for 10 minutes. Add 5x protein loading buffer (BL502A, Biosharp) and treat with 95 degrees for 10 minutes.

Prepare 10% polyacrylamide gel. Add 4μL of protein marker(26616, Thermo) and 8μL of sample in sequence. Use 60V for 30 minutes of electrophoresis and then switch to 110V for 60 minutes of electrophoresis. The mold transfer condition is 200mA for 120 minutes. Block with 5% nonfat dry milk for one hour. Add antibody solution (1:10000 for anti-GAPDH(Cat No:AC002, Abclonal), 1:1000 for Anti-TLR4(Cat No:A17436, Abclonal),Anti-p-PI3K (Cat No:4228T, CST),Anti-PI3K (Cat No:A4992, Abclonal), Anti-p-AKT (Cat No:AP1259, Abclonal),Anti-AKT(A20799, Abclonal)) and incubate at 4 degrees overnight. The next day, the membrane was washed and incubated with HRP-conjugated secondary (BL001A, BL003A, Biosharp) antibody for 1 hour. Photographs were taken using Millipore luminescent fluid and a Li-COR chemiluminescence imaging system. Use stripping buffer to strip off the antibody from the membrane, allowing new antibodies to be incubated after blocking.

Cytolysosomal pH Fluorescence Staining

RAW264.7 cells were seeded in confocal dishes and cultured in DMEM high-glucose medium containing 10% FBS for 24 hours. The experimental group was stimulated by adding materials ($CaCO_3$, ZIF-8, Alpha-MOF), and the control group was added with the same amount of PBS. After incubating at 37°C for 3 hours, discard the medium, rinse gently three times with 1 mL of PBS, and absorb the liquid in the dish as much as possible. Add 200 μL of PDMPO lysosomal pH probe (1:1000, 40768ES50, YEASEN) and protect from light for 5

minutes. Photographs were taken using a fluorescence confocal microscope (excitation light at 375 nm and 440 nm).

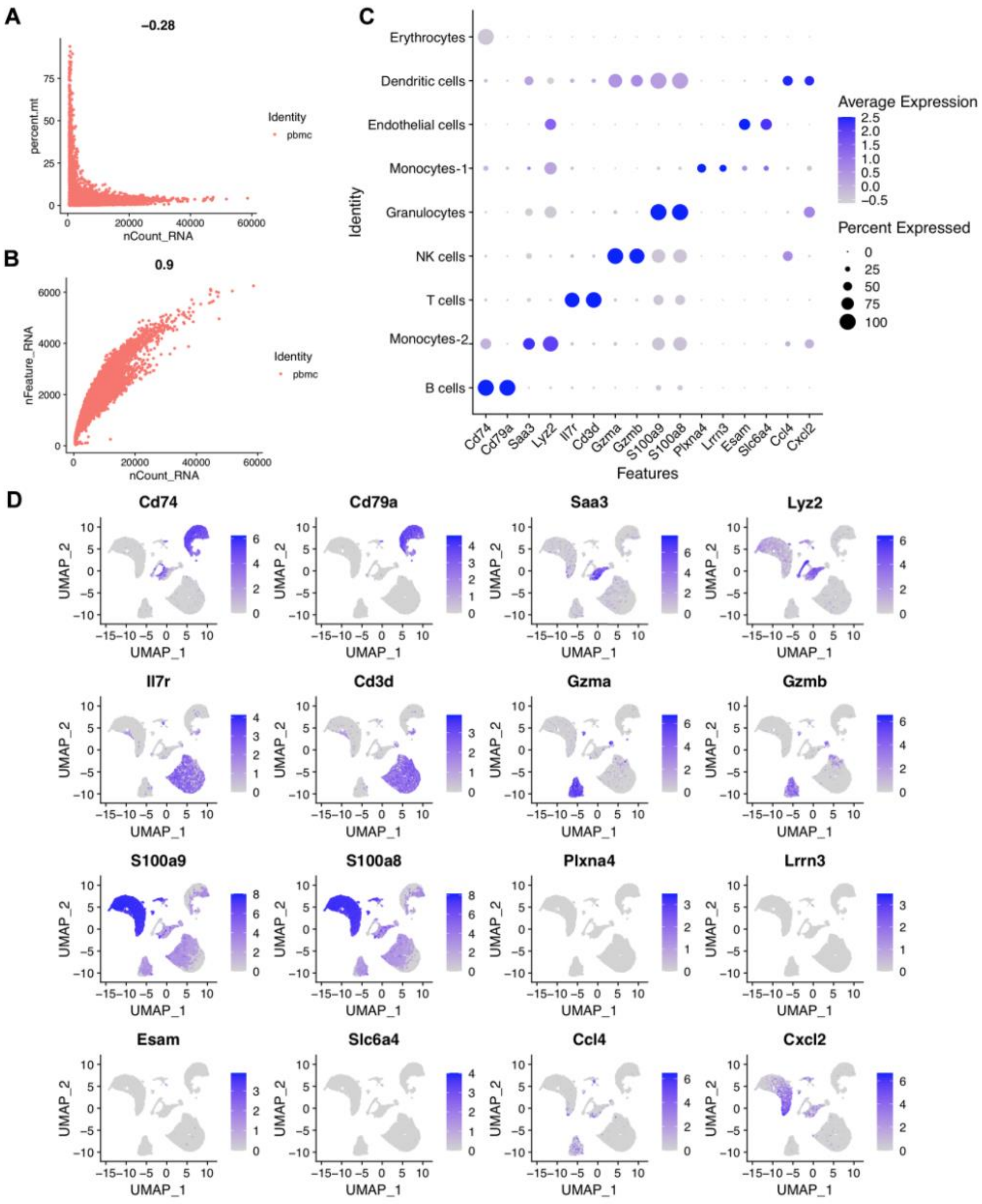

Figure S1. (A)-(B)Scatter plots showing the relationship between Count and Percentage of mitochondria genes as well as Count and Feature. For quality control, cells with a percentage of mitochondrial genes below 10% and between 200 and 5000 genes detected were retained.

(C) Dot plot showing representative marker genes for cell populations.(D) UMAP plots of total cells showing the marker genes of individual cell populations.

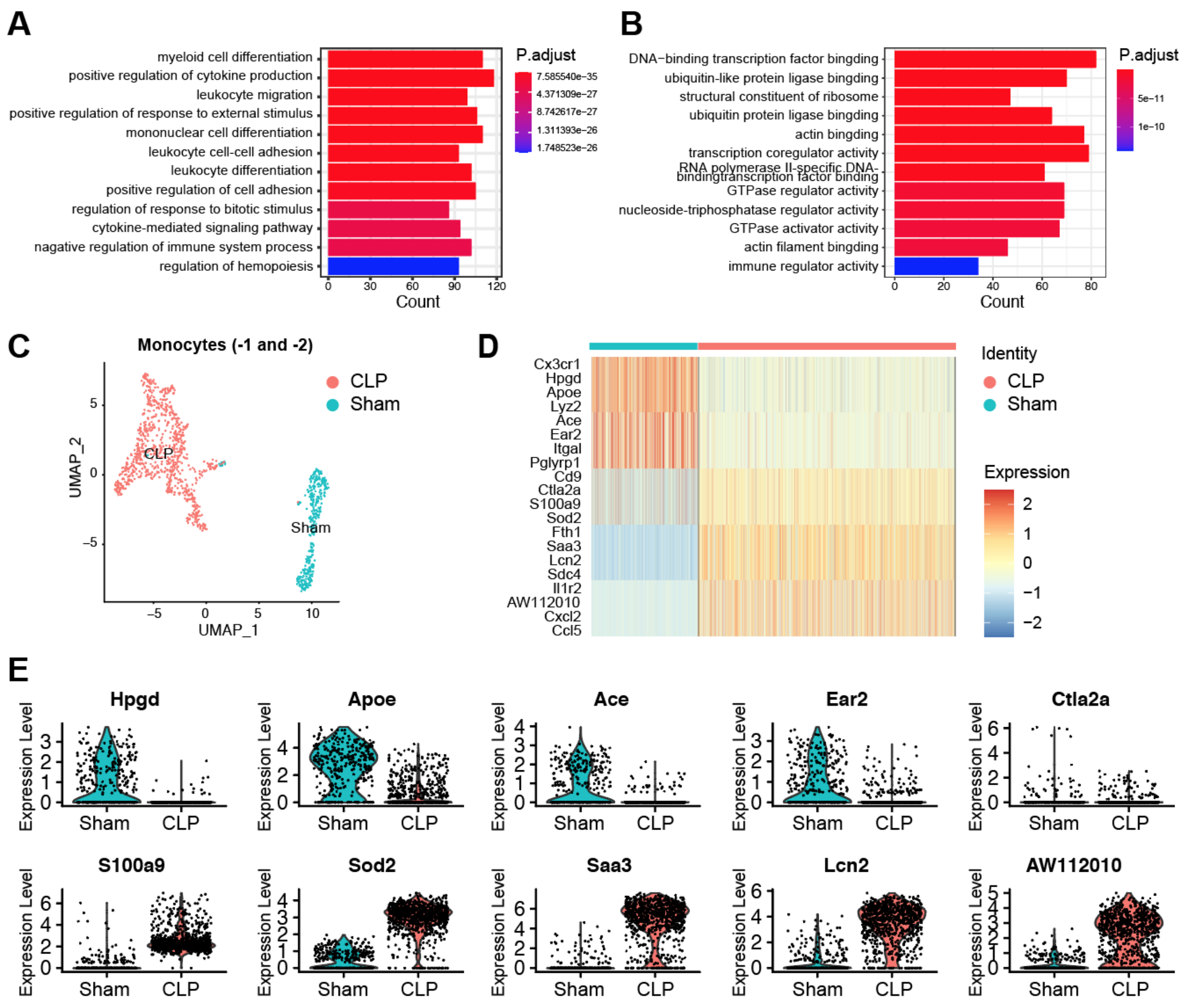

Figure S2. (A)-(B) The bubble charts show the representative rich gene ontology (GO) term analysis of monocyte population, including biological process (D: BP) and molecular function (E: MF). (C) UMAP plot of monocytes only [cluster Monocyte -1 and -2 in Fig.1B]. (D) Heatmap of differentially expressed genes (DEGs) sorted by group, showing the top 10 DEGs. (E) Violin plots of differentially expressed genes for each group of monocytes.

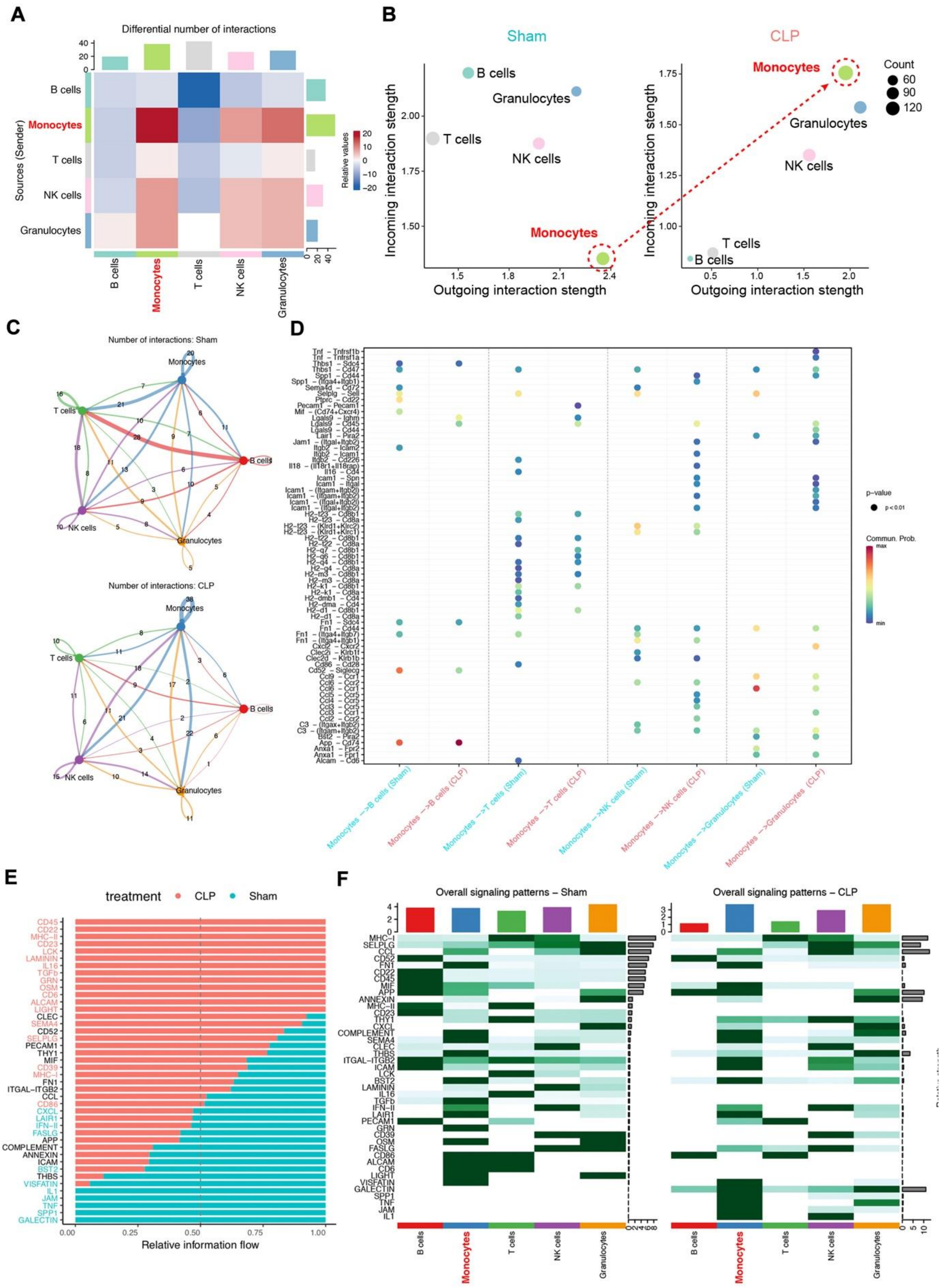

Figure S3. (A) Heatmap depicting the number of all possible interactions between the clusters analyzed. (B) Communication probabilities of outgoing signaling and incoming signaling corresponding to each cell group. (C) Number of significant ligand-receptor pairs between any pair of two cell populations. (D) Histogram showing conserved and specific signaling

pathways in two groups of monocytes. (E) Signal flow patterns of two groups of monocytes. (F) Dot plot of chemokine-ligand-receptor interactions between monocytes and other cells. Shown are indicators that are highly expressed in the CLP group.

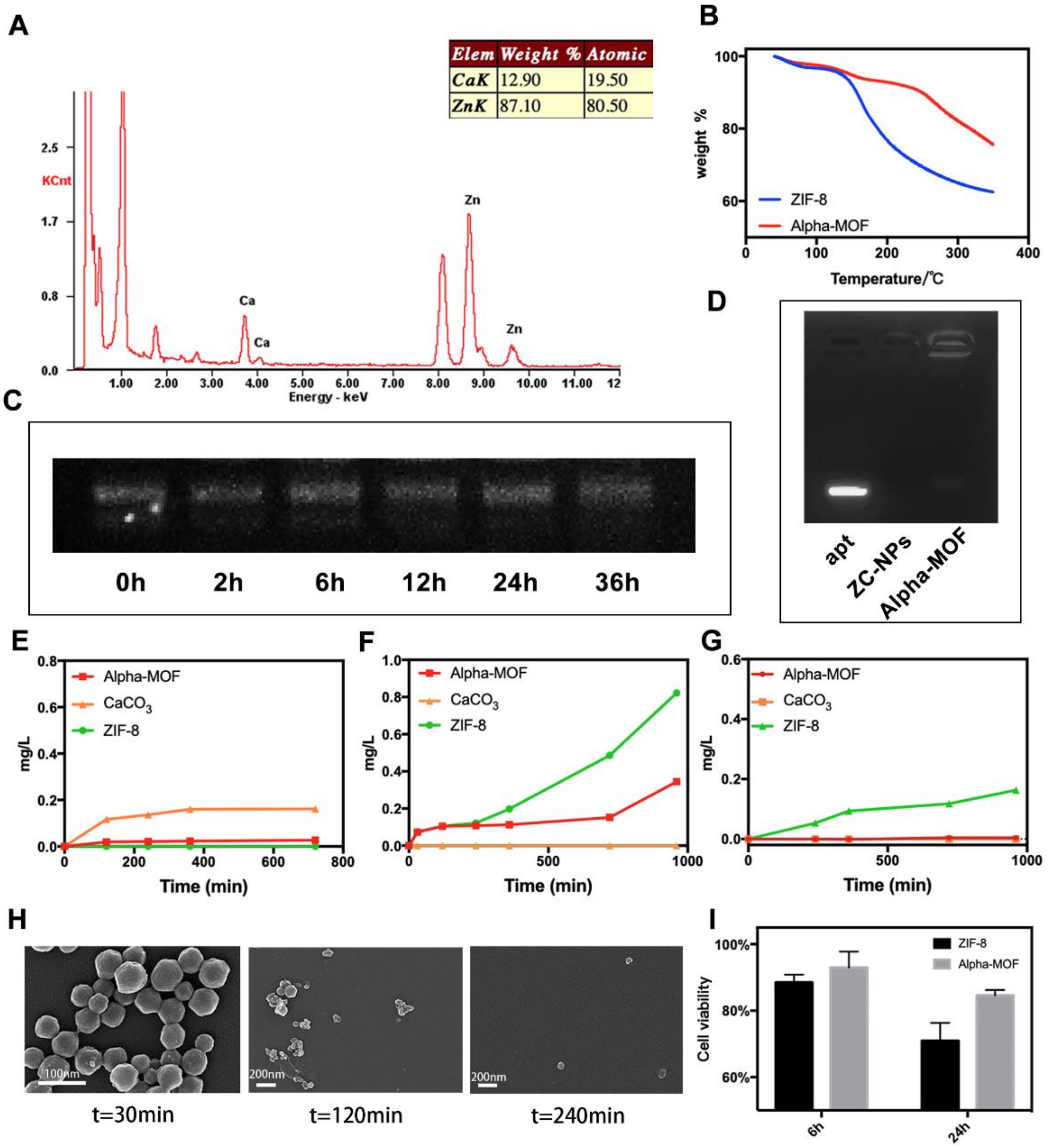

Figure S4. (A) Elemental Energy Spectrum Analysis of Alpha-MOF. (B) Thermogravimetric Analysis of Alpha-MOF and ZIF-8. (C) Long-term stability electrophoresis of aptamers in PBS containing 10% serum at 37°C. (D) Electrophoresis images of aptamers attached to nanoparticles. (E) Calcium ion release curve of the nanomaterials at pH=7.4. (F) Zinc ion release curve of the nanomaterials at pH=5.5. (G) Zinc ion release curve of the nanomaterials at pH=7.4. (H) SEM images of the gradual degradation of Alpha-MOF at pH=5.5. (I) Cytotoxicity assay of ZIF-8 and Alpha-MOF using CCK8 method(n=3).

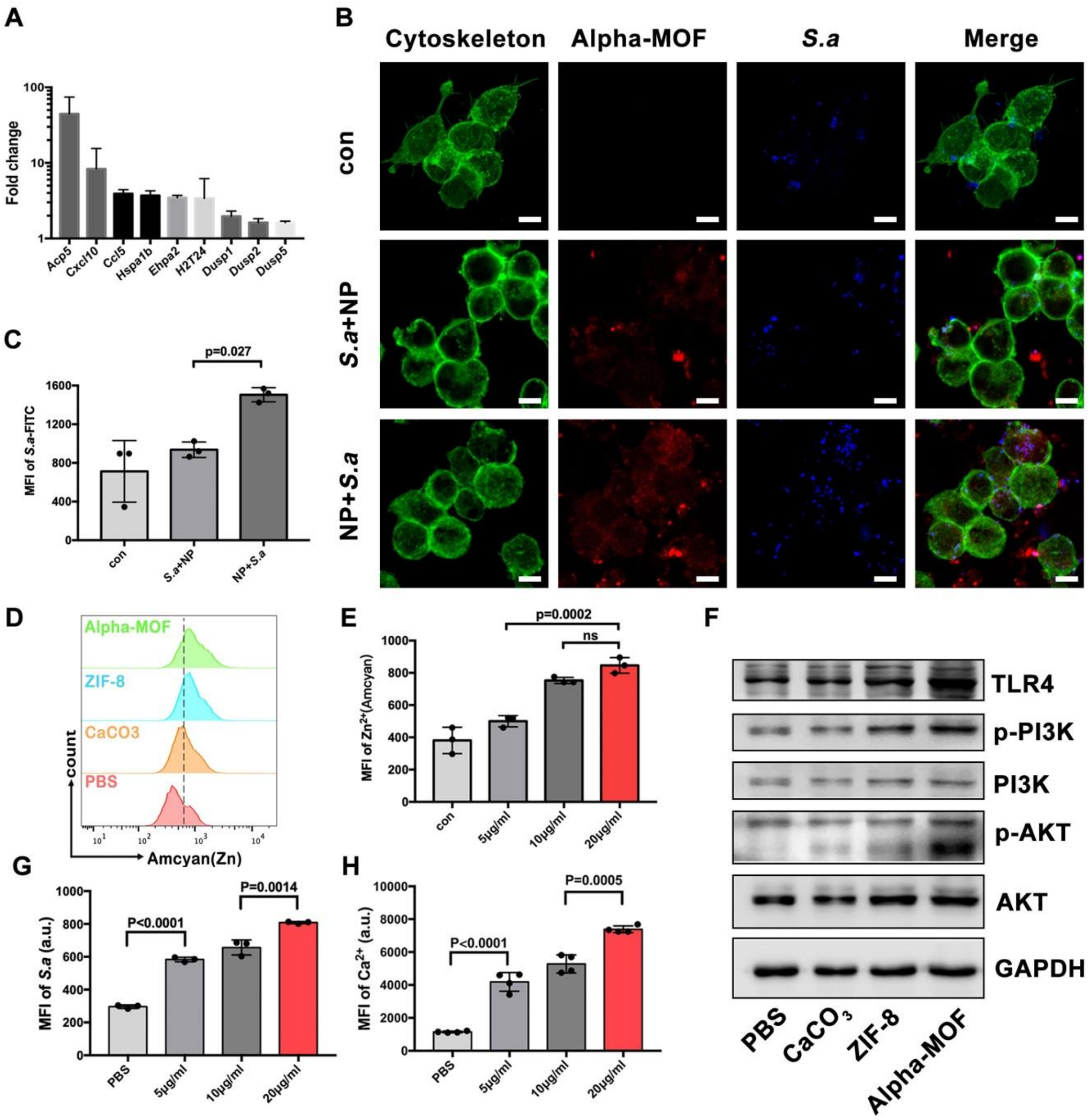

Figure S5. (A) qRT-PCR validation of highly expressed genes from RNA transcriptome sequencing(n=3). (B) Fluorescence confocal images show that the order of adding Alpha-MOF and bacteria affects the phagocytosis of bacteria.(C) Flow cytometry analysis of the effect of the order of adding Alpha-MOF and bacteria on the phagocytosis of bacteria(n=3). (D)- (E) Flow cytometry analysis of intracellular zinc ion content after adding various materials/concentrations(n=3). (F) Exploring phagocytosis-related pathways or protein expression by Western Blot. (G) Flow cytometry analysis of the effect of adding different amounts of Alpha-MOF to cells on their phagocytosis of bacteria(n=3). (H) Effect of adding different contents of Alpha-MOF to cells on intracellular calcium content by flow cytometry (n=4).

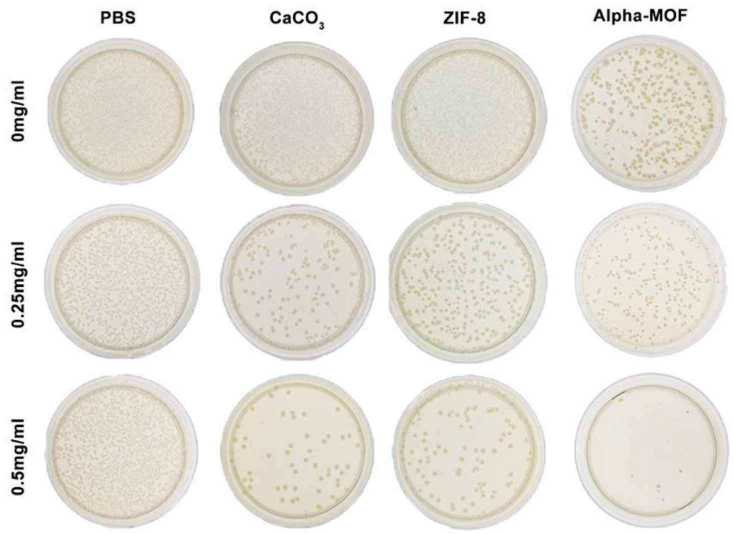

Figure S6. Plate photo of bacterial content in adding PBS, CaCO3, ZIF-8, and Alpha-MOF to cell.

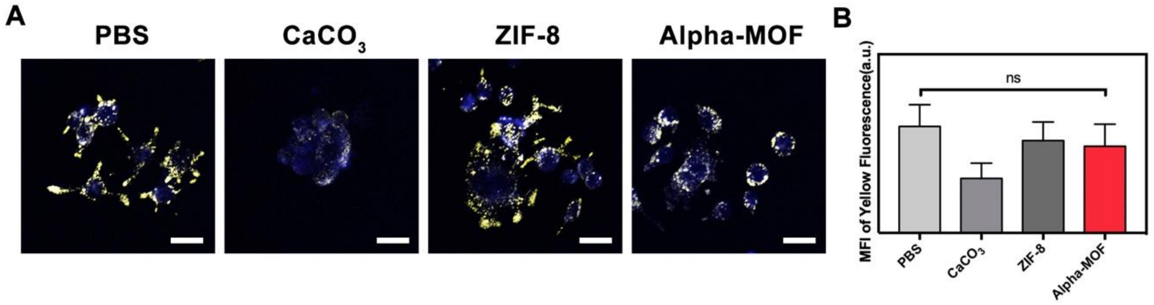

Figure S7. (A) Fluorescence confocal images showed changes in lysosomal pH of cells after the addition of different materials. The more prominent the yellow in the image, the more acidic the lysosome is. (B) Quantitative Statistics of Yellow Fluorescence Intensity in Figure I (n=3).

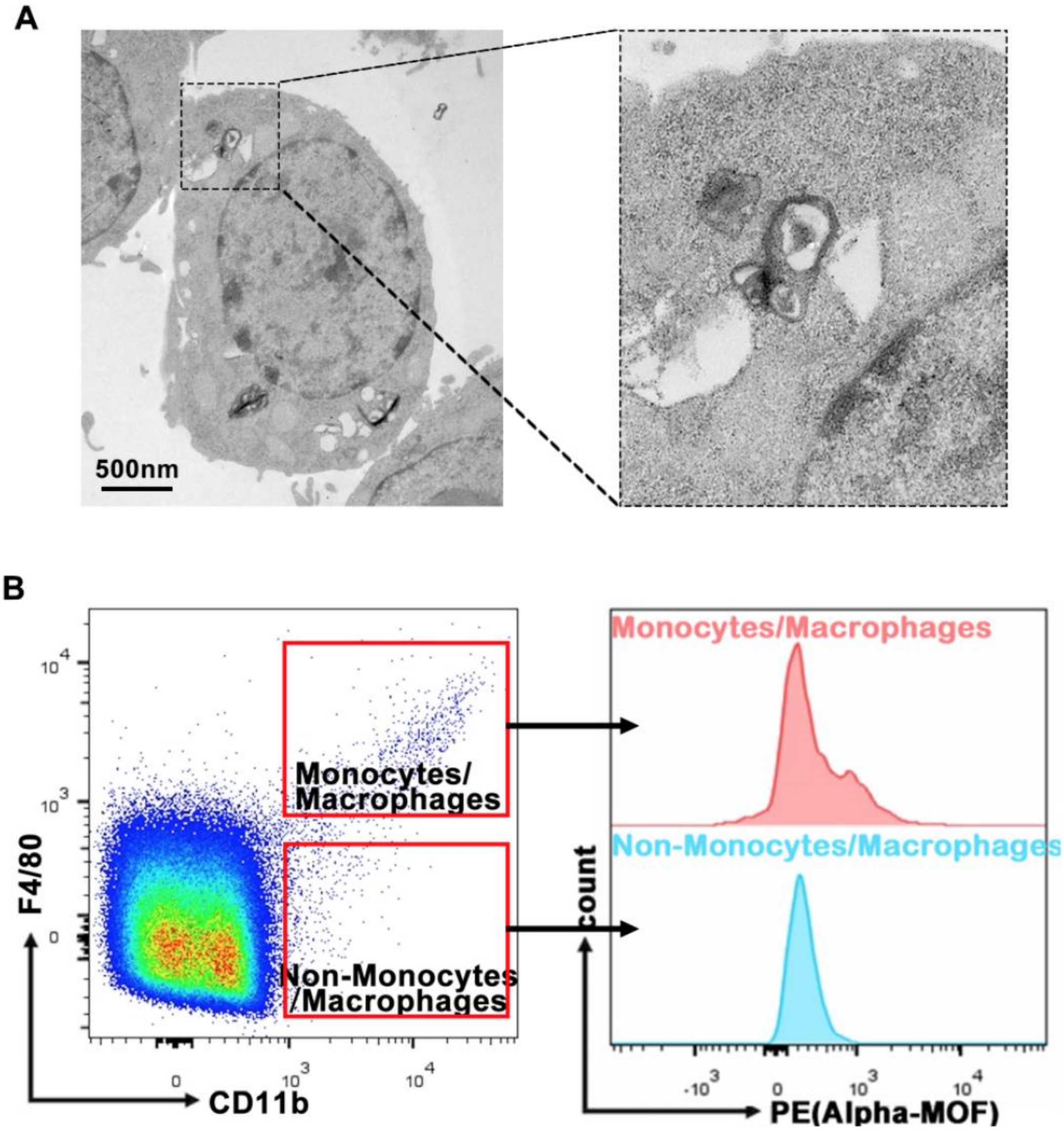

Figure S8. (A) Cellular TEM shows internalization of Alpha-MOF. (B) Flow cytometry to detect the ratio of monocytes/macrophages to non- monocytes/macrophages in the blood targeted by Alpha-MOF.

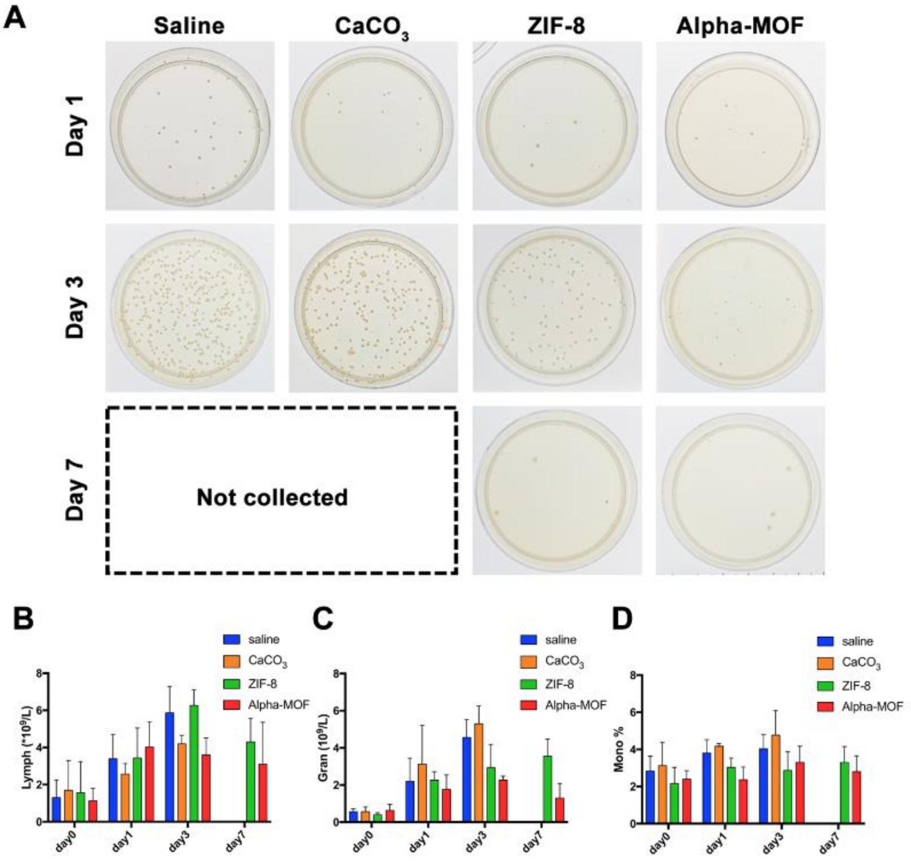

Figure S9. (A) Plate photo of bacterial content in blood of septic mice. (B)-(D) Blood routine test of septic mice, followed by the number of lymphocytes, the number of granulocytes and the percentage of monocytes(n=3).

A

	Saline	ZIF-8	$CaCO_3$	Alpha-MOF
WBC 10^9/L	5.01±3.78	7.46±1.37	6.28±1.95	5.47±1.60
NEU %	60.47±30.02	17.90±10.57	11.47±8.05	20.07±11.87
Lymph %	34.23±31.57	72.73±0.81	70.17±4.18	70.20±6.26
Mon %	3.62±1.20	8.50±11.60	17.45±5.14	7.77±12.59
EOS %	1.26±1.17	0.13±0.12	0.03±0.06	0.97±1.34
BAS %	0.43±0.14	0.73±0.12	0.88±0.65	1.00±0.30
NEU 10^9/L	2.35±0.92	1.24±0.63	0.77±0.70	1.22±0.99
Lymph 10^9/L	2.45±3.50	5.43±0.93	4.42±1.43	3.82±0.96
Mon 10^9/L	0.15±0.06	0.74±1.07	1.04±0.22	0.32±0.51
EOS 10^9/L	0.04±0.01	0.01±0.01	0.00±0.01	0.07±0.10
BAS 10^9/L	0.02±0.02	0.05±0.01	0.05±0.04	0.05±0.00
RBC 10^{12}/L	6.00±0.98	8.19±0.62	7.92±0.83	7.77±0.60
HGB g/L	98.00±19.08	143.00±5.20	136.67±10.21	135.67±9.71
HCT %	33.90±6.14	46.83±0.81	47.27±4.01	47.20±3.82
MCV fL	56.40±1.68	57.33±3.52	59.73±1.91	60.70±0.87
MCH pg	16.30±0.69	17.50±0.69	17.27±0.65	17.47±0.15
MCHC g/L	288.67±4.04	305.67±5.77	289.33±3.21	288.00±3.61
RDW %	14.13±0.32	13.00±0.00	13.50±0.26	13.63±0.40
PLT 10^9/L	716.33±194.58	1172.33±188.22	1091.00±246.27	1131.33±247.33
MPV fL	5.57±0.46	5.40±0.17	5.60±0.36	5.43±0.21
PDW %	14.47±0.12	14.7±0.17	14.73±0.12	14.63±0.06
PCT %	0.40±0.14	0.63±0.08	0.61±0.09	0.61±0.11

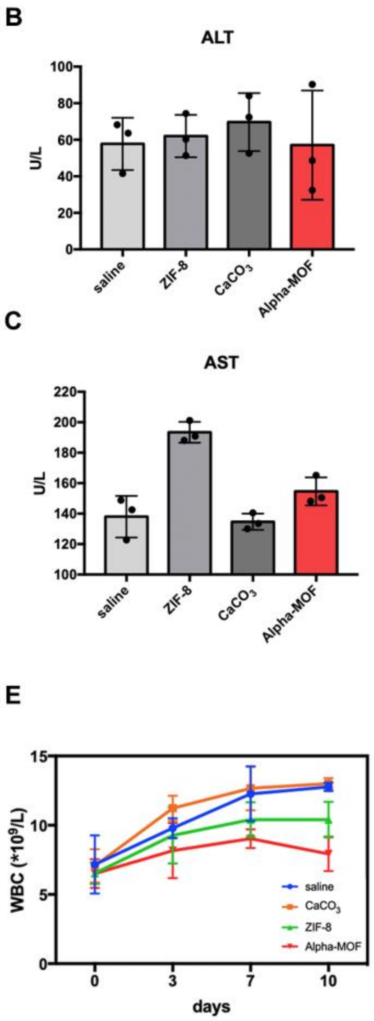

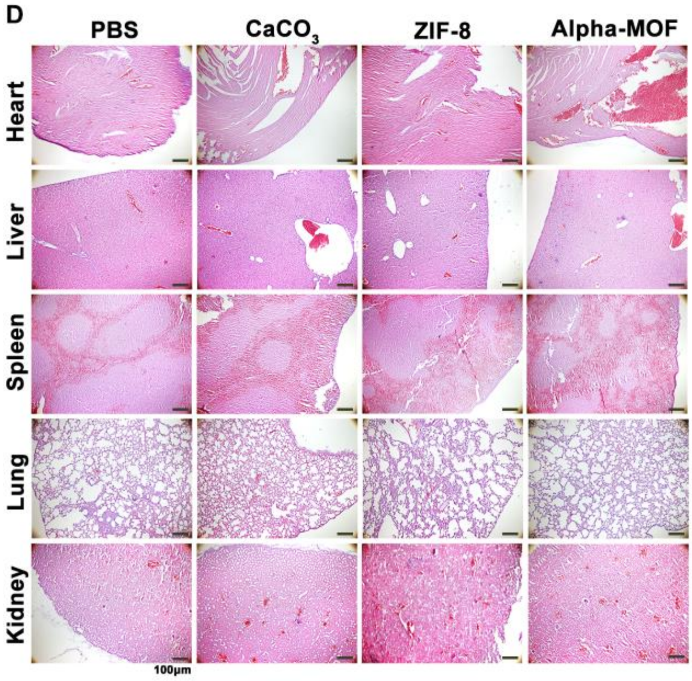

Figure S10. (A) Blood routine test of healthy mice after infusion of material into tail vein(n=3). (B)- (C) Blood biochemical indexes were detected after infusion of materials into the tail vein of healthy mice(n=3). (D) HE staining was performed on the main organs of healthy mice after the material was injected into the tail vein. Scale bars: 200 μm.(E) Statistical chart of the hemolysis test. (F) Routine blood leukocyte count in local infection model mice(n=3).

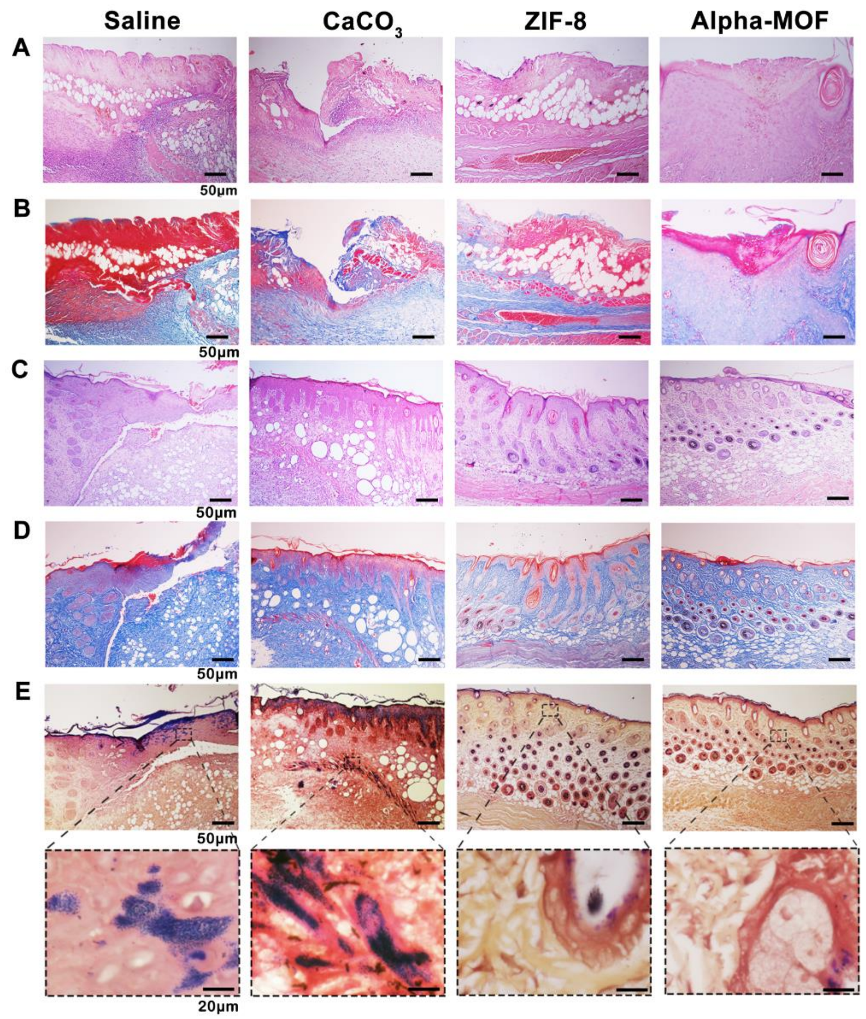

Figure S11. (A) HE staining pictures of local infection sites in mice (day 3). (B) Masson staining pictures of local infection sites in mice (day 3). (C) HE staining pictures of local infection sites in mice (day 7). (D)Masson staining pictures of local infection sites in mice (day 7). (E) Modified Gram-stained pictures of local infection sites in mice (day 7).

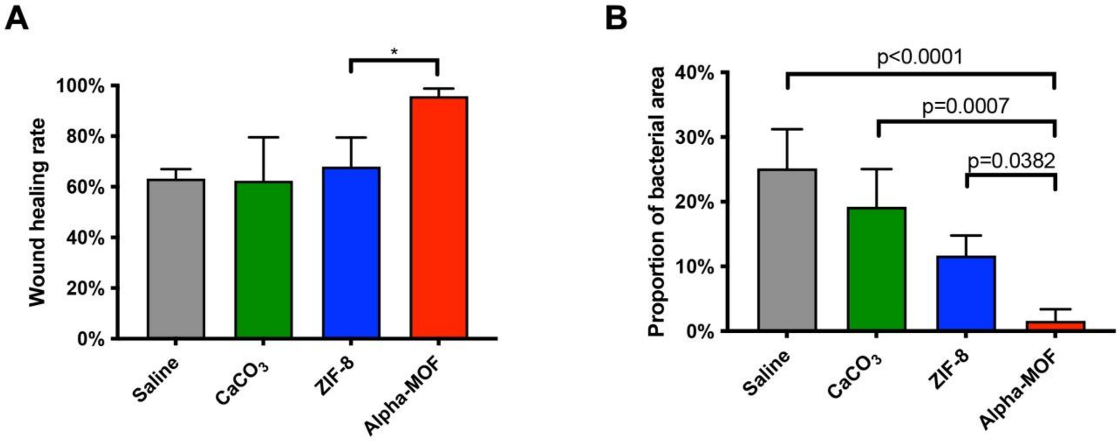

Figure S12. (A) Statistical analysis of wound size change after material treatment in mouse local infection model (Fig. 5H). (B) Statistical analysis of Modified Gram-stained images (Fig. 5I) in mice at day 3.
